# Use of implementation science to advance family planning programs in low- and middle-income countries: A systematic review

**DOI:** 10.3389/fgwh.2022.1038297

**Published:** 2022-12-06

**Authors:** Colin Baynes, Petrus Steyn, Caroline Soi, Aneth Dinis, Stelio Tembe, Hedieh Mehrtash, Manjulaa Narasimhan, James Kiarie, Kenneth Sherr

**Affiliations:** ^1^ Department of Global Health, University of Washington, Seattle, WA, United States; ^2^ Department of Sexual and Reproductive Health and Research, UNDP/UNFPA/UNICEF/WHO/World Bank Special Programme of Research, Development and Research Training in Human Reproduction, Geneva, Switzerland; ^3^ Department of Epidemiology, University of Washington, Seattle, WA, United States; ^4^ Department of Industrial and Systems Engineering, University of Washington, Seattle, WA, United States; ^5^ The National Directorate of Public Health, Ministry of Health of Mozambique, Maputo, Mozambique

**Keywords:** implementation science, strategy, research, family planning, low- and middle-income country

## Abstract

**Objective:**

As environmental and economic pressures converge with demands to achieve sustainability development goals, low- and middle-income countries (LMIC) increasingly require strategies to strengthen and scale-up evidence-based practices (EBP) related to family planning (FP). Implementation science (IS) can help these efforts. The purpose of this article is to elucidate patterns in the use of IS in FP research and identify ways to maximize the potential of IS to advance FP in LMIC.

**Design and methods:**

We conducted a systematic review that describes how IS concepts and principles have been operationalized in LMIC FP research published from 2007–2021. We searched six databases for implementation studies of LMIC FP interventions. Our review synthesizes the characteristics of implementation strategies and research efforts used to enhance the performance of FP-related EBP in these settings, identifying gaps, strengths and lessons learned.

**Results:**

Four-hundred and seventy-two studies were eligible for full-text review. Ninety-two percent of studies were carried out in one region only, whereas 8 percent were multi-country studies that took place across multiple regions. 37 percent of studies were conducted in East Africa, 21 percent in West and Central Africa, 19 percent in Southern Africa and South Asia, respectively, and fewer than 5 percent in other Asian countries, Latin America and Middle East and North Africa, respectively. Fifty-four percent were on strategies that promoted individuals' uptake of FP. Far fewer were on strategies to enhance the coverage, implementation, spread or sustainability of FP programs. Most studies used quantitative methods only and evaluated user-level outcomes over implementation outcomes. Thirty percent measured processes and outcomes of strategies, 15 percent measured changes in implementation outcomes, and 31 percent report on the effect of contextual factors. Eighteen percent reported that they were situated within decision-making processes to address locally identified implementation issues. Fourteen percent of studies described measures to involve stakeholders in the research process. Only 7 percent of studies reported that implementation was led by LMIC delivery systems or implementation partners.

**Conclusions:**

IS has potential to further advance LMIC FP programs, although its impact will be limited unless its concepts and principles are incorporated more systematically. To support this, stakeholders must focus on strategies that address a wider range of implementation outcomes; adapt research designs and blend methods to evaluate outcomes and processes; and establish collaborative research efforts across implementation, policy, and research domains. Doing so will expand opportunities for learning and applying new knowledge in pragmatic research paradigms where research is embedded in usual implementation conditions and addresses critical issues such as scale up and sustainability of evidence-informed FP interventions.

Systematic Review Registration: https://www.crd.york.ac.uk/prospero/, identifier: CRD42020199353.

## Introduction

Decades of experience make it clear that interventions to improve family planning (FP) policies and programs can be effective at low cost in relatively controlled environments, be they in externally supported projects, short-term studies or small-scale public initiatives (1–3). Yet, integrating them and promoting their scale-up and sustained effectiveness in health systems remains a challenge, particularly in low- and middle-income countries (LMIC) (4–7). Implementation science (IS) is the study of methods to promote the systematic uptake of research findings and other evidence-based practices (EBP)^1^ into routine practice (8) in order to improve the coverage, quality, sustainability, and effectiveness of health services (9). Although embedding research in FP programs is not new to LMIC (10), applying IS as a formal discipline in global health initiatives is nascent, and can contribute to large-scale successes needed to achieve universal healthcare coverage (UHC) and the Sustainability Development Goals (SDGs) (11–14). Recognition of this potential illuminates a gap in understanding how well and widely IS is employed and how its principles, concepts and methods could be applied to increase impact of FP programs in LMIC (15, 16).

IS builds on multiple research traditions that have their own set of core disciplines, audiences, and methodologies (17). Many of these have been applied to evaluate and enhance FP programs. For example, operations research has been a mainstay of strategies to introduce and improve FP services in health systems (18, 19). Organizational science has helped guide the transfer of project innovations to the public sector (20), evaluate policy implementation (21, 22) and systematically introduce new contraceptive technologies (23, 24). The recommendations of the International Conference on Population and Development (ICPD) in 1994 led to research on FP integration within a wider spectrum of SRH services, quality of care, client perspectives, and community empowerment (25–29). In the past 15 years, FP researchers have focused on implementation of EBP and scale-up (30).

The parallel histories of contributing research disciplines, siloed funding and infrastructure, and the lack of standards for adapting and reporting on their use perpetuate debates over the definitions and boundaries of IS (31, 32). Yet, there is convergence that IS is the systematic use of research methods to improve EBP coverage, delivery, sustainment and spread throughout complex systems. IS emphasizes attention and adaptation to local context, stakeholders, local care resources and meaningful end-user engagement (17, 33). Furthermore, there is consensus on prominent features of IS, including adequate description of the EBP and the implementation problem targeted. Adaptation and targeting of implementation strategies vis-à-vis these underlying circumstances should be clear, including the actors who enact the strategy (34–36), so as to promote effective tailoring of intervention and evaluation approaches (37–39). The importance embedding research in the “real world” and using feedback loops that facilitate uptake of findings in “real time” is widely understood, as is the need to study the effect of contextual factors on outcomes and processes. Research teams should include diverse stakeholders and empower decision-makers to act as both “research producers” and the consumers of new knowledge (31, 40–42). IS methods ought to evaluate the effects of interventions to improve the adoption, delivery and sustainability of EBP, balance focus on processes and outcomes, and be flexible to contextual shifts (8, 43, 44). IS emphasizes responsiveness to knowledge needs of target audiences and engagement of stakeholders in evidence generation and use (45–47). Understanding the degree to which these facets of IS are employed across the spectrum of FP research is critical for determining ways to guide evidence-informed strengthening of policies and programs.

We review how the concepts, methods, and principles of IS have been applied to the adaptation, specification, and evaluation of implementation strategies that aim at maximizing the potential of EBP related to FP in LMIC. The goal of this review is to inform decision-making on how IS can be better applied to enhance the integration, delivery, spread and sustainability of FP interventions in these settings. This will have the potential to lead to downstream improvements in SRH of women, girls, gender-diverse individuals, their families, and communities.

## Methods

### Research questions

We performed an integrated, mixed method systematic review of relevant literature of IS studies about FP in LMIC (PROSPERO registration CRD42020199353), following the “Preferred Reporting Items for Systematic Reviews and Meta-Analyses” (PRISMA) guidance (48). To define our research question, we employed the “PICCO” framework, i.e., participants, interventions, comparisons, contexts, and outcomes (PICCO) (Supplementary Material 1). The review addresses two research questions:

1. How have users of IS applied IS concepts and constructs in the design and execution of implementation strategies to enhance the performance of FP-related EBPs in LMIC?
2. How have users of IS employed theories, methods, and principles of IS in implementation research on strategies to enhance FP-related EBP delivery in LMIC?

### Study search and title-abstract review

Studies for the review were identified via a literature search of PubMed, Scopus, Cochrane, Web of Science, EMBASE and CINAHL using search terms on implementation strategies, implementation outcomes, geographic area of focus and year of publications (January 2007 until December 2021)^2^. The identified studies were uploaded into the systematic review software, Covidence, for review. Three researchers (CB, CS and HM) independently reviewed titles and abstracts to assess their eligibility for full-text review. There were four criteria for this: (1) an explicit focus on FP-related EBP (e.g., contraceptive method, individual behavior or attitude related to FP, a delivery or management intervention that emphasizes FP, or a policy or large scale program that emphasizes FP); (2) having taken place in an LMIC in the above timeframe (determination of LMIC status was based on the World Bank classification); (3) specification of an implementation problem, issue or challenge around the performance of the EBP that was the target of an intervention and; (4) evaluation of implementation-related outcomes associated with an implementation strategy, including contraceptive uptake. Studies were deemed eligible for full-text review if two researchers agreed that the above criterion were met. When disputes arose, a third reviewed the study abstract and the majority opinion was put into effect. Systematic reviews were not considered for full text review. However, researchers did review the reference sections of pertinent systematic reviews to obtain the titles of possibly eligible studies, and such studies were included in the title/abstract review. In addition, studies were only eligible for full text review if they were written in English.

### Full-text review

Full-text analysis was completed in a computerized data extraction form in Redcap software. There were three steps to the full text review: (1) assessment of whether the research was of sufficient quality according to research quality assessment tools for quantitative and qualitative studies produced by the Effective Public Healthcare Panacea Project and Critical Appraisal Skills Program (49, 50); (2) assessment of whether the studies were sufficiently detailed vis-à-vis questions in Boxes 1 and 2 and, of the studies that met the above quality criteria; (3) descriptive analysis of salient IS descriptors grouped around the two research questions (Boxes 1, 2). Five researchers contributed to the first stage of the full text review (CB, CS, HM, AD and ST). Studies admitted into the descriptive analysis were reviewed by two researchers. Disagreements were resolved through discussion with coauthors until consensus was reached.

BOX 1 **Key characteristics for reporting implementation strategies**

1. The underlying implementation or decision-making issue that needs to be addressed to improve adoption, implementation and sustainability of an EBP.
2. Evidence on the intervention and contextual information that supports its selection to address the issue.
3. Use of knowledge or data on context to adapt, or customize, the strategy.
4. The actors that lead and contribute to the strategy, emphasizing co-creation of strategies across sectors and/or disciplines.
5. The beneficiary targets and modifiable factors the strategy aims at changing to best address the issue.
6. Activities or components the strategy comprises.
7. The sequence and amount of each activity or component to be implemented.

BOX 2 **Key characteristics for reporting implementation research**

1. Use of theories, models and frameworks for guiding research and evaluation.
2. Description of how findings support better adoption, implementation and sustainment of evidence-based practices (i.e., to address the implementation or decision-making issue).
3. Dual focus on process and outcomes.
4. Incorporation of contextual analysis on strategy design, implementation processes and outcomes.
5. Positioning of research within decision-making, evidence-use frameworks, responsive to local information needs.
6. Collaboration between decision-makers, implementation leaders and researchers on research plans and evidence-use.
7. Embedded in realities of implementing health systems, organizations and communities with a focus on sustainment.

To classify implementation strategies, we drew upon the WHO health systems building blocks framework (51). We added other descriptors for this classification because they applied to many studies in the full text review. Standardized classification taxonomies have been developed to foster an evidence base on effective implementation strategies. These range from clinically-oriented categorizations to those focused on improving systems performance (52, 53). In our review, we built upon the latter, and classified strategies using criteria informed by the Consolidated Framework of Implementation Research (CFIR) (54), Expert Recommendations on Implementing Change Project (36), and, especially, the taxonomy applied by Leeman et al. (2017) (35, 55). Within each grouping of strategies, one researcher (CB) analyzed studies more finely to identify trends in terms of actor roles, strategy components and modifiable factors they sought to address.

To synthesize findings on implementation research, researchers sought consensus on the research objectives that each selected study prioritized. Those called out in this paper were selected based on reviewer pairs' shared opinion on the strength of their example in this regard. To assess the use of implementation outcomes, we drew upon the “implementation outcomes framework” delineated by Proctor et al. (2013) (56). We calculated descriptive statistics and cross-tabulations to describe the sample's distribution and identify patterns in the adaptation and use of IS to enhance FP-related EBP in LMIC health systems.

Finally, we conducted an in-depth qualitative synthesis of a subset of studies with most complete reporting of salient IS characteristics. Studies included in the descriptive analysis were eligible for the qualitative synthesis if they met at least five of the criteria described in Table 5 or if three reviewers agreed that the study met three criteria in Table 5 and was particularly exemplary vis-à-vis one of them.

## Results

### Descriptive analysis

#### Background and evidence-based practices

**Figure 1:**
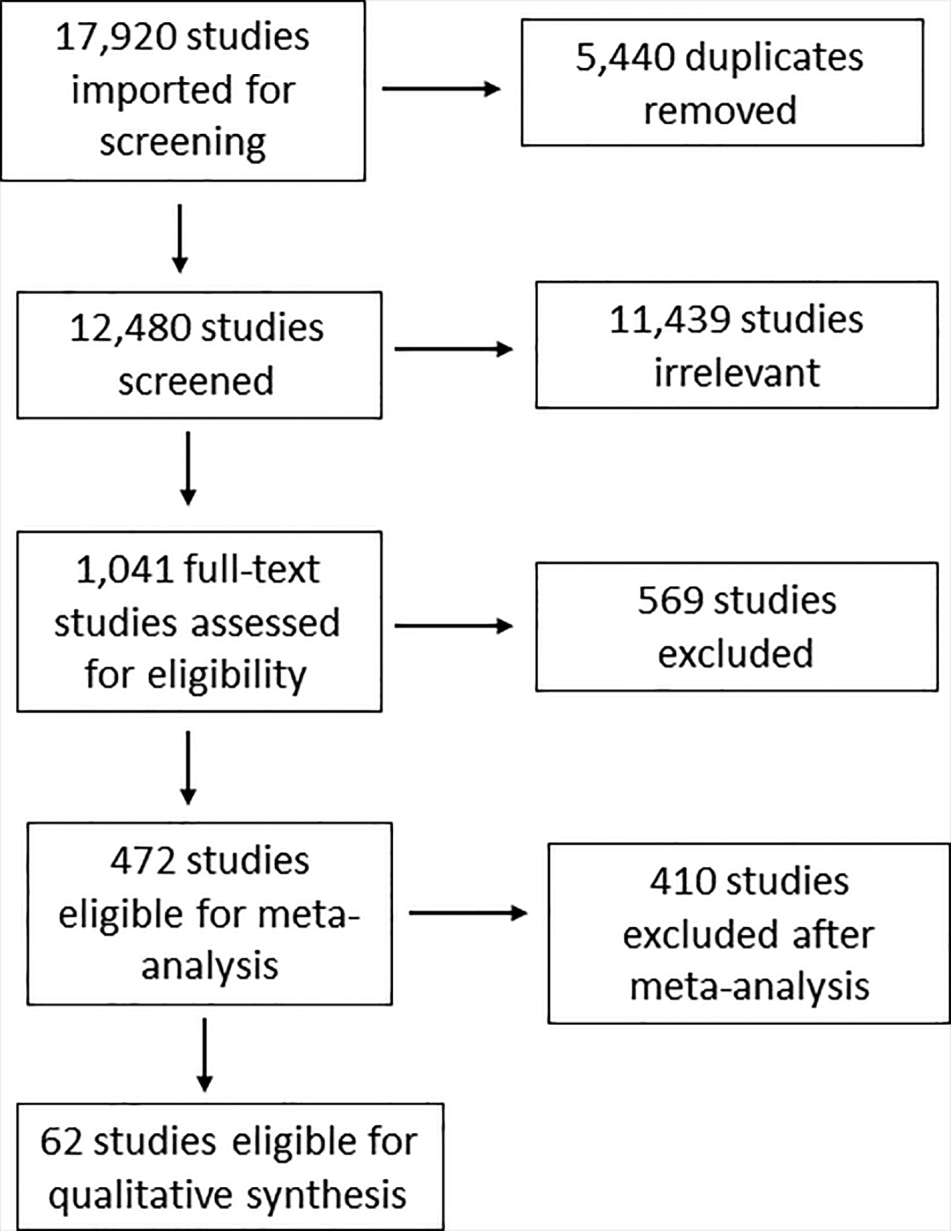
PRISMA flowchart delineating the process used to conduct the systematic review.

Figure 1 is the PRISMA flow chart of our systematic review. The initial search generated 17,920 studies, of which 5,440 were duplicates and removed. Of the remaining 12,480 potentially eligible studies, 1,041 (8%) were selected for full-text review. In the descriptive analysis were included 472 studies of which 238 considered contraceptive methods as the only or primary EBP (the object that the implementation strategy sought to improve coverage, quality and/or sustainability of), and 51 focused on an individual FP-related behavior or attitude.

Approximately 39 percent of studies focused on a service delivery intervention (*n* = 129), management or organizational level intervention (*n* = 30, 6%) or policy or large-scale program (*n* = 24, 5%) as the EBP. In 72 percent of studies, EBP targeted women of reproductive age (*n* = 340), while fewer targeted couples (*n* = 75, 16 percent) and adolescents/youth (*n* = 57, 12 percent). Over 30 percent of studies fully reviewed took place in East Africa (*n* = 176, 37 percent), followed by West and Central Africa (*n* = 102, 21 percent), Southern Africa (*n* = 90, 19 percent), South Asia (*n* = 90, 19 percent), China and other Asia (*n* = 24, 5 percent), Latin America (*n* = 21, 4 percent), Middle East and North Africa (*n* = 17, 4%), and Eastern Europe/ former Soviet Republics (*n* = 2, <1 percent). Ninety-two percent of studies (*n* = 432) were carried out in one region only, whereas 8 percent (*n* = 40) were multi-country studies that took place across multiple regions.

#### Implementation strategies

Implementation strategies constitute the “how to” component of change in routine practice (34). In examining the articles, we found that 62 percent (*n* = 291) definitively reported who led implementation strategies, and the balance reported that strategies were led by some combination of delivery system and support system actors. Thirty-six percent of studies (*n* = 164) targeted eligible contraceptive users only as the object of the intervention, whereas 56 percent (*n* = 266) targeted potential users and other member(s) of the service delivery system. Similarly, most implementation strategies focused on promoting individuals' use of FP services (*n* = 257, 54 percent), while fewer prioritized implementation objectives (Table 1).

**Table 1:** Specification of implementation strategies—prevalence of key intervention descriptors.

	*N* (%)
Salient implementation strategy activities are described*	
*WHO Health System Building Blocks Classification*	
Service delivery improvements (e.g., service quality improvements, strengthening of counseling, integration of FP with other services)	233 (49)
Health workforce ((re)deployment, (re)orientation health workers or capacity building beyond the level of routine training in the manner of task shifting or an innovative approach to packaging or coordinating training)	193 (40)
Health information systems	23 (5)
Technological solutions to expand the reach of FP information and services or address limitations in the method mix available	113 (24)
Financing and social protections	68 (14)
Leadership and governance	11 (2)
*Other*	
Community engagement (i.e., delivery or support system actor elicits participation of community health workers, leadership, or groups to implement the EBP)	159 (34)
Social and behavioral change communication	62 (13)
**Leader of implementation strategy is explicitly defined**	
Delivery system (i.e., members of the health system responsible for delivering routine care)	199 (42)
Support system (i.e., implementation partner organizations that build the capacity of delivery systems to deliver EBP)	84 (18)
Synthesis and translation system actors (i.e., organizations that manage new knowledge, disseminate and promote uptake of evidence during implementation)	8 (2)
Combination of delivery system and support system.	181 (38)
**Primary target(s) of implementation strategy is explicitly defined** *	
Users of FP services	266 (56)
Health workers	193 (41)
Community health workers (including volunteers or groups based in communities)	88 (19)
Private sector health workers	56 (12)
Local government authorities (e.g., district health management team)	40 (8)
Men or couples	40 (8)
National level policymakers	24 (5)
**Objectives of implementation strategies are clear (the foremost objective of the implementation strategy is…)** **^a^** **^,b^**	
To enhance or promote individuals' use, understanding of and uptake of contraception and FP services	257 (54)
To enhance or promote the implementation or delivery of FP-related EBP	94 (20)
To enhance or promote systems' adoption of FP-related EBP	90 (19)
To enhance or promote the sustainment of FP-related EBP	6^b,c^ (1)

*Multiple classifications can apply per study.

^a^ More than one descriptor can apply but provided are our assessment of the principal objective for each strategy.

^b^ 25 studies featured strategies that prioritized a combination of objectives equally.

^c^ Although only 7 studies prioritized enhancing the sustainability of FP-related EBP, sustainability issues were addressed by 56 strategies to some extent (49 giving it less emphasis than adoption and implementation).

Of the strategies reviewed fully, more than half (*n* = 251, 53 percent) were classified as “integration strategies”, reflecting programs' targeting of factors in individuals' spheres, service delivery settings or the wider health system and community environment that impede and/or facilitate use of FP methods. The second most-common classification was of “capacity building” strategies, which are led by support system actors that target delivery systems to enhance individual and team motivation, self-efficacy, and skills to implement EBP (18 percent, *n* = 84) (57).

We classified 14 percent (*n* = 68) of implementation strategies as scale up strategies, i.e., strategies led either by support system actors or high-level actors in delivery systems (e.g., Ministries of Health) with the goal of getting multiple settings to implement specific FP-related EBP. Only 8 percent (*n* = 40) of studies featured implementation process strategies that were enacted by those working within FP service delivery systems and pertain to processes or activities that implementation teams perform to plan, select, and integrate FP-related EBP into routine practice. Whereas capacity building and integration strategies aim at promoting better adoption and implementation of specific EBP, implementation process strategies target how well teams execute processes that apply to EBP generally (including EBP bundles) (58). Finally, 6 percent (*n* = 29) of studies were classified as having dissemination strategies, which utilize communication and awareness-raising activities to target the attitudes, knowledge, and intentions of audiences to accept and adopt FP-related EBP (59). See Table 2. Less than half of the studies (42 percent, *n* = 199) clarified the temporal sequence of delivery of intervention components. While most studies identified implementation outcomes that strategies sought to change (80 percent, *n* = 378), a minority (11 percent, *n* = 50) provided a detailed theory of change, causal pathway or conceptual model. Few studies provided information on the amount, duration or intensity of implementation that was required, or delivered, to achieve outcomes (14 percent, *n* = 68) (156–158).

**Table 2:** Classification of implementation strategies assessed in studies included in full text review (*n* = 472) using the taxonomy of leeman et al. (2017) (35).

Integration strategies		
*Target/purpose: To address factors that facilitate or hinder the adoption and integration of an FP-related EBP in a setting to promote higher levels of use of the practice (focus on user and implementer).*		
**Type of strategy found in literature**	**Examples**	
Technological solutions to reposition FP within lifeworld of user, and enhance the opportunities, intentions, abilities to use FP.	mHealth FP education/ awareness messages, referral to services; mHealth reminder systems for current users, telemedicine counselling to support contraceptive self-care.	McCarthy et al (2018, 2019); Smith et al (2017); Reiss (2017, 2019); Harrington (2019); Bates et al (2018); McConnell, et al. (2018) (60–67).
Changes in the service delivery environment or efforts to enhance the organizational capacity or climate to increase opportunities and motivations to use the EBP.	For users, better counseling, or access to information (e.g., leaflets), co-location of FP counseling or methods in other health service delivery settings. For implementer, job aids, decision-support tools, financial or material inputs to improve readiness, coaching and mentoring, customized tools to enhance referrals.	Dulli, et al. (2019); Tran (2018; 2019); Church et al. (2015); Warren, et al. (2012); Ojanduru, et al. (2018); Biswas, et al. (2017); Grossman, et al. (2013); Farouk-Eslamou et al. (2013); Chabikuli, et al. (2009) (68–77)
Revision or expansion of health worker roles, including involvement of non-traditional actors, to better situate the FP vis-a-vis users, enhance opportunities, motivations, and abilities to use.	Deployment of community-health volunteers or workers to make FP services convenient; recruitment of SRH focused peer educators in schools and non-traditional health settings; configuring an FP service within the private sector to increase access; self-administered contraception, including injectable contraception.	Bates, et al. (2019) Bacqui et al. (2018); Mudiope et al. (2017); Cover (2018); Burke (2018; 2018); Hernandez, et al. (2018); Mullany, et al. (2010) (78–86)
Social and behavioral interventions aimed at removing social and cultural barriers and enhance individuals' confidence and self-efficacy vis-à-vis the EBP.	Formation of peer groups to promote an enabling environment for SRH care seeking; targeting gatekeepers; gender transformative interventions to advance autonomy of women and girls, and gender-diverse individuals, male engagement, couples’ communication	Huda, et al. (2019); Bapolisi, et al. (2020); Challa (2019); Chirwa-Kambole, et al. (2020); Subramanian, et al. (2018) (87–91).
Pro-poor financial interventions to offset the costs and promote affordability of FP use.	Targeted voucher schemes.	Ovugi, et al. (2017); Ali, et al. (2019); Atukunda et al (2019); Bellows et al (2011) (92–95)
**Capacity building strategies**		
*Target/purpose: To enhance individuals’ motivation and capability to implement an EBP (focus on implementers).*		
Task sharing to different levels of care and cadres.	Training and deploying community health workers to deliver FP; training women to self-inject DMPA-SC; task sharing tubal ligation to health officers; private sector provision of postabortion care.	Reiss et al. (2018); Jacinto et al. (2016); Cover et al. (2017); DiGiorgio et al. (2018); Nuccio, et al. (2016); Ogu et al. (2012) (96–102)
Building management capacity.	Training in business management, strengthening and support for organizational structures to promote sustainability.	Ugaz et al. (2017); Canning, et al. (2016); Mugore, et al. (2016); Karra et al. (2019); Hackett et al. (2020) (103–107)
Training and post-training follow up vis-à-vis a specific EBP.	In-service trainings in postpartum FP and postabortion FP; training community health workers in FP; trainings of health care workers in rights-based approaches and incorporating them in service delivery.	Kiemtore et al. (2019); Cooper et al. (2020); LeFevre et al (2018); Hardee et al. (2019); Wendot, et al. (2018) (108–112).
Strategies to strengthen or improve upon existing capacity building approaches or trainings.	Online training approach for postpartum IUD insertion; peer mentoring as a strategy for capacity building; digital health training package in community-based distribution of FP	Zafar et al (2019); Ndwiga et al (2014); Limaye et al (2020) (113–115)
**Scale up strategies**		
*Target/Purpose: To enhance motivation and capacities to integrate a specific EBP or package of EBP into practice across multiple settings; to enhance the ability of EBP coverage to expand.*		
**Type of strategy found in literature**	**Examples**	
Results-based incentivization of adopting EBP packages throughout national health systems.	Pay for performance schemes led by national figures that engage local leaders in policies that contain EBP, reward facilities or districts for EBP implementation, encourage use of rewards to improve service delivery, and public performance benchmarking.	Zeng et al (2018, 2018); Nahimana et al. (2016); Friedman et al. (2016) (116–119)
Policy driven initiatives led by NGO to strengthen human resource at large scale vis-à-vis specific EBP.	Training and technical support to expand youth friendly LARC and postabortion FP; integrating non-communicable disease interventions into existing national CHW platform;	Fikree et al. (2019); Samuel et al. (2016); Dunbar et al. (2018) (120–122)
Establishment of social franchise networks, including targeted voucher schemes, to expand EBP adoption and use.	Promote and enforce service provision and quality standards; expand networks through capacity building; financial incentives to remain in network and offset care seeking costs.	Prata et al. (2013); Bellows et al. (2017); Pereira et al. (2015); Liu et al. (2018); Azmat et al. (2013) (123–127).
Top-down policy implementation led by Ministries of Health.	Adolescent health programming and implementation of the national RMNCH policies in India; public-sector financing of maternal health and family planning in Mexico; task sharing in Burkina Faso.	Barua et al. (2020); Taneja et al. (2019); Avila-Burgos et al. (2016); Millogo et al. (2019) (128–131).
Projects that transition from NGO initiatives to national programs achieving larger scale	Incorporation of ExpandNet framework for scaling up into NGO activities that progressively achieve wider scale, increasingly through the structures and resources of national public health systems.	Igras et al. (2014); Keyonzo et al. (2015); Aichatou et al (2016); Mai et al. (2019): Ntabona et al. (2021) (132–135)
**Implementation process strategies**		
*Purpose/target: How well teams execute processes to select, adapt and integrate EBP or packages of EBP into routine practice; processes are not exclusive to specific EBP but are/can applied to this effect with respect to any EBP.*		
Stakeholder engagement, use of evidence, planning.	National stakeholder planning of costed implementation plans; effectiveness of guidelines and their promotion to improve laws and policies; district-level health planning and priority setting; advocacy frameworks to promote policy changes.	Lipsky et al (2016); Shilton, et al. (2019); Chitama et al. (2011); Gichane et al. (2018) (136–139)
Execution of processes aimed at optimizing performance of EBP already in practice.	Strengthening of organizational networks to improve cross-level referral in municipal health system; models for putting service integration into place and maximizing access; interventions to strengthen the functionality of supply chain; “plan, do, study, act” processes to strengthen service integration and quality.	Thomas et al (2016); Faye et al (2015); Cavallaro et al. (2016); Hasselback et al. (2017); Krug et al. (2020); Tawfik, et al. (2014) (140–144)
Use of research as a strategy to facilitate EBP adaptation and adoption in health systems.	Engaging stakeholders in using data to make decisions on scale up FP services; pilot research as advocacy; formative and process evaluation to guide develop models for service improvements.	Byrne et al. (2012); Binanga et al. (2016); Spring et al. (2016); Milford et al. (2018) (145–148)
**Dissemination strategies**		
*Purpose/target: To build societal-level or systems-wide awareness of and knowledge about specific EBP, attitudes in favor of the EBP, intentions to adopt the EBP.*		
User and/or gatekeeper targeted	Spread awareness, understanding and intention to use, or support others' use, of contraception or other desirables SRH behaviors.	Burke et al. (2014); Okigbo et al. (2015); Beaudoin et al (2016); Atagame et al. (2017); Babalola et al. (2017) (149–153)
Health care worker or manager targeted	Spread awareness, understanding and intentions to adopt best practices for implementing FP.	LeMay et al. (2012); Kraft et al. (2018) (154, 155).

#### Implementation research

Core to IS is the use of theories, models, and frameworks to gain insight on why and to what extent implementation succeeds or fails (159). These have centered on three aims: (1) describing, or guiding, the process of translating evidence into practice; (2) understanding and/or explaining what influences implementation outcomes; and (3) evaluating implementation and the effectiveness of strategies (160). Initially, this review sought to quantify the frequency with which studies explicitly used a common IS theory, model, or framework, and found that only 3 percent (*n* = 14) studies did so, of which six called out “process models”, five determinants frameworks, and three evaluation frameworks. An additional forty-four articles referenced other theories, models, or frameworks, mostly concerning health behavior change, to articulate theories of change.

To understand underlying influences of theoretical perspectives, reviewers classified studies by whether they addressed specific research objectives, recognizing that more than one descriptor could apply to a single study (Table 3). Sixty-three percent (*n* = 301) of studies evaluated implementation effectiveness in terms of uptake of a health behavior, including service utilization, 31% (*n* = 147) sought to understand the barriers and facilitators that influenced whether a strategy was successful, 24% (*n* = 113) to examine processes of integrating EBP within a setting, 12% (*n* = 58) to assess the spread of EBP to target audiences, 10% (*n* = 48) to understand whether a strategy was being implemented according to a plan and if implementation could improve, and 3% (*n* = 12) how research participants and stakeholders can learn from findings and be empowered to act.

**Table 3:** Classification of research objectives prioritized in fully reviewed studies (*n* = 472).

Type of research objective	*N* (%)	Examples
Determine effectiveness of implementation vis-à-vis intended outcomes.	301 (64)	Phillips et al. (2012); Baqui et al. (2018); Carmichael et al. (2019) (80, 161, 162)
Understand the factors that influenced whether a strategy was successful.	147 (31)	Mutemwa et al. (2013); Myers et al. (2018); Hackett et al. (2020) (103, 163, 164)
Examine processes of integrating EBP within a setting.	113 (24)	Tessema et al. (2019); Zulu et al. (2019); Milford et al. (2018) (145, 165, 166)
Assess the spread of EBP to target audiences	58 (12)	Awoonor-Williams et al. (2013); Gold et al. (2017); Adenini et al. (2018) (167–169)
Understand whether a strategy was being implemented according to a plan and if implementation could improve.	48 (10)	Rawlins et al. (2013); Agha et al. (2010); LeFevre et al (2018) (109, 170, 171)
How research participants and stakeholders can learn from findings and be empowered to act.	12 (3)	Butler et al. (2020); Boydell et al. (2018); Tran et al. (2018); Chhoun et al. (2019) (172–175)

Sixty-three percent (*n* = 299) of studies used quantitative methods only, while the remainder either drew upon qualitative methods only (19 percent, *n* = 87) or were mixed method (18 percent, *n* = 86). Of the quantitative studies, 24 percent (*n* = 91) were randomized trials, 69 percent (*n* = 265) employed quasi-experimental designs, and 7 percent (*n* = 26) triangulated cross-sectional data with qualitative methods. Of the qualitative studies, 24 percent (*n* = 42) were formative studies to guide adaptation of implementation strategies, 35 percent (*n* = 61) described or explained processes, and 41 percent (*n* = 71) obtained data to explain quantitative outcomes.

We classified studies according to outcome type, distinguishing outcomes of effectiveness (rows 9–10 in Table 4) from implementation outcomes (rows 1–8), recognizing that many studies assess more than one (Table 4). Overall, 72 percent (*n* = 341) evaluated an implementation outcome, whereas the remainder (28 percent, *n* = 131) reported only behavior or service utilization outcomes. Forty-eight percent (*n* = 228) were classified as “implementation-effectiveness hybrid designs” as they measured both outcome types (44). Of these, 73 percent (*n* = 166) placed greater emphasis on the effectiveness variable, and 24 percent (*n* = 113) focused solely on implementation outcomes.

**Table 4:** Variables measured by fully reviewed studies.

		Quantitative measures (%)	Qualitative measures (%)
**Implementation Variables (56)**			
1	Acceptability	74 (16)	123 (26)
2	Adoption	50 (11)	19 (4)
3	Appropriateness	6 (1)	25 (5)
4	Costs	38 (8)	0 (0)
5	Feasibility	40 (8)	104 (22)
6	Fidelity	46 (10)	17 (4)
7	Penetration	41 (9)	7 (1)
8	Sustainability	11 (2)	14 (3)
**Effectiveness Variables**			
9	Health or behavioral outcome	188 (40)	29 (6)
10	Service utilization	297 (62)	11 (2)

Table 5 features examples of studies that are related to the following implementation research themes: (1) focus on processes and outcomes, (2) incorporation of contextual analysis, (3) responsiveness to evidence demands and fitness to purpose, (4) multidisciplinary and multisectoral collaborations, and (5) embeddedness of research in real world implementation scenarios.

**Table 5:** In-depth review of iS studies with more complete reporting of salient iS characteristics.

Criteria	Examples	
**Focused on processes and outcomes**		
Evaluation of processes to explain outcomes and/or how implementation can be improved.	Explain factors that affected the implementation and effectiveness of PPIUD initiative; Assess effect of integrating HIV risk counseling on injectable contraceptive uptake; Elucidate contextual factors affecting quality and uptake of FP during immunization services; Process evaluation of reproductive health voucher program; Tracking user interactions with providers in an mHealth intervention to identify ways to refine implementation; Process evaluation of quality improvement collaborative and implementation barriers and facilitators focused on community-based distribution of FP.	Hackett et al. (2020); Barden O’Fallon et al. (2020); Nelson et al. (2019); Abuya et al. (2012); Smith et al. (2017); Kim et al. (2019) (63, 104, 177–180).
Monitor deviations from implementation protocols, i.e., lapses in fidelity to implementation plans.	Monitoring implementer fidelity to counseling protocol during a PPIUD intervention trial; Mapping planned vs actual implementation of a FP-focused social accountability intervention; Assessment of fidelity to design of community-based distribution program; Monitoring of FP stock outs and investigating lapses in supply chain management; Tracking success rate of a mHealth intervention at connecting callers to health workers to receive tele-counseling; Comparing facilities' sustainment of integrated SRH/FP service delivery; Monitoring fidelity to original community-based health planning and services intervention package during scale up.	Karra et al (2019); Boydell et al. (2019); Hasselback et al. (2017); Mayhew et al. (2017); Phillips et al. (2012) (107, 162, 176, 180, 181)
Determine whether interventions improve or cause change in implementation outcomes.	Effect of digital training tools on quality of FP counseling; changes in the quality of FP service delivery during and after a donor-funded program; Measuring change in adolescent perceptions of the acceptability of modern contraception after an mHealth education intervention; Assessing effect of post-training provider coaching mentoring on quality of abortion-care services; Effect of business-skills and clinical capacity building intervention on management and FP service delivery in the private sector; Effect of network strengthening meetings on HIV-FP referral processes.	Limaye et al. (2020); McCarthy et al. (2019, 2018); Benson et al. (2017); Ugaz et al. (2017); Thomas et al. (2016) (104, 116, 141, 182, 183)
**Incorporation of contextual analysis**		
Explain how implementation strategies were adapted and/or customized according to local programmatic contexts.	Political economy analysis to guide strategy on how civil society organizations should adapt work plans and promote SRH in local political context; Qualitative formative research to develop integrated community-based HIV prevention and SRH services for women that inject drugs; Participatory methods to build an mHealth intervention for female entertainment workers; Qualitative formative research to inform design of private sector FP training and referral system; Participatory action research to develop postpartum FP interventions; Health information needs assessment to develop a national model for SRH knowledge exchange; Use of organizational capacity assessments to guide strategy on adding long acting reversible contraceptives to the method mix; Embedding qualitative research in scale up to adapt rural implementation models for urban settings.	Butler et al. (2020); Ayon et al. (2019); Chhoun et al. (2019); Bates et al. (2019) Tran et al. (2018); LeMay et al. (2012); Sprockett (2016); Adongo et al. (2014) (78, 155, 172–174, 184–186).
Measure and/or explain how contextual factors affected implementation and outcomes.	Examine how different contexts trigger mechanisms of acceptability with respect to the delivery of FP services in childhood immunization settings; Understand barriers and facilitators to implementation-effectiveness of female FP volunteers; Provider perceptions of the constraints and facilitators to using national guidelines for FP service delivery; Systems analysis to identify context-specific constraints to FP service delivery to guide scale up planning.	Webster et al. (2021); Marston et al. (2020); Tessema et al. (2019); Church et al. (2015); Byrne et al. (2012) (68, 147, 165, 187, 188)
**Research that is fit for purpose and responsive to local demands for knowledge**		
Research design and methods are flexible, adaptive to emergent information needs and context.	Use of human-centered design to develop and evaluate context-specific youth-driven solutions to unmet need for FP; Triangulation of intervention fidelity monitoring, rapid provider surveys and documentation review to guide mid-project course corrections of community-based distribution program; Systematic adaptation and use of routine M&E to guide scale up of Standard Days Method; Embedded data quality assurance and support for real-time data analysis for decision-making vis-à-vis key performance indicators to guide mid-program action of a performance-based financing project; Use of behavioral economics to iteratively refine postabortion FP counseling quality improvement interventions.	Atchison et al. (2018); Doyle et al. (2019); Hernandez et al. (2018); Igras et al. (2017); Nahimana et al. (2016); Spring et al. (2016) (79, 118, 134, 189–191)
Positioned within a local decision-making framework, driven by demand for knowledge on local implementation challenges and solutions.	Government commissioned evaluation of adolescent sexual and reproductive health policy implementation to guide improvements and scale up; Embedded implementation research to guide FP task sharing policy development and scale up; Evaluation of national results-based financing program to guide decisions on scale up; Pilot research as advocacy to guide introduction and scale up of Sayana Press; Review and assessment of compliance with policy implementation milestones to guide scale up of community-based primary health care and family planning.	Barua et al. (2020); Friedman et al. (2016); Binanga et al. (2016); Awoonor-Williams et al. (2013) (119, 130, 146, 166)
**Multidisciplinary and/or multidisciplinary collaboration**		
Emphasizes co-creation of implementation research plans by practitioners, researchers, decision-makers.	Donor-implementer-policy collaboration to guide task sharing for FP services; Pragmatic trial of public-sector led community-based health systems strengthening (includes FP); Government and research partner collaboration to embed a cluster randomized trial into the roll out of a public sector led reproductive and child health systems intervention that evaluates impact on government set endpoints; Government led evaluation of RMNCH policy implementation strategy using routine data from supervisions across multiple states (included FP); Design and evaluation of supply and demand-side interventions urban reproductive health initiative.	Millogo et al. (2019); Lauria et al. (2019); Carmichael et al. (2019); Taneja et al. (2019); Aichatou et al. (2016) (128, 131, 162, 192, 193).
Describes engagement of practitioners, decision-makers and/or communities in use of evidence or translation of knowledge to practice.	Translating evidence in policy changes on community-based distribution of injectable contraception; Using program knowledge to develop and refine strategies for scaling up throughout wider health system; Government adaptation and use of WHO FP guidelines and tools to formulate laws, policies, and strategies; Developing a model for integrating sexual and reproductive health services with HIV prevention and care; Explore the policy and systems factors that shape district-level priority-setting and planning processes for FP and MCH interventions'.	Gichane et al., (2019); Mai et al (2019); Shilton et al. (2021); Milford et al. (2018); Kraft et al. (2016); Keyonzo (2015); Chitama et al. (2011) (132, 133, 136, 137, 139, 145, 154)
**Embedded in the “real world”**		
Implementation is led by local delivery systems without help from a support system actor or help from a local support system actor.	Assessing a referral model for better integration HIV and FP services using routine data; Using a behavioral economics approach to address contraceptive discontinuation; Integrating community health assistant driven sexual and reproductive health services in community health systems; Investigation of whether changes in national health expenditure patterns were consistent with relevant policy objectives; Small clinic network staff strategically plan and execute intervention to integrate FP services with HIV care and treatment.	Chabikuli et al. (2019); Karim et al. (2019); Zulu et al. (2018); Mulubwa et al. (2020); Avila-Burgos et al. (2016); Phiri et al. (2013); Tweya et al. (2018). (69, 129, 166, 194–197)
Generates knowledge on how to promote sustainment of implementation strategy.	The sustainability of the impact of the urban reproductive health initiative; Integration and sustainment of community-based adolescent sexual and reproductive health interventions; Sustainment of the impact of the community health and family planning project; Institutionalizing PPIUD implementation as a lasting, integral component of maternal health care; Using demand-side financing strategies to make high quality FP services available at scale.	Speizer et al. (2019, 2019); Zulu et al. (2018); Phillips et al. (2012); Canning et al. (2016); Azmat (2014) (105, 161, 165, 198–201)

Of the 341 studies that measured implementation outcomes, 14 percent (*n* = 49) measured changes in implementation variables over time, and 34 percent (*n* = 117) measured both outcomes and processes. Sixteen percent (*n* = 53) of studies reported whether implementation processes deviated from implementation plans. Only one-third (*n* = 156) of studies provided information on the context of the EBP and if the implementation strategy was adapted to it. Thirty-one percent (*n* = 147) reported how contextual factors affected implementation and outcomes (202).

We found that 46 percent (*n* = 215) of studies clearly articulated the target audience and that 29 percent (*n* = 136) link results with recommendations that address implementation problems. Eighteen percent (*n* = 85) of studies indicated how they were situated within local decision-making frameworks and address stakeholder-expressed implementation challenges and demands for new knowledge. Only 10 percent (*n* = 48) of studies reported how research designs and methods were adapted to emergent information needs and context, and 31 percent (*n* = 148) reported that researchers involved stakeholders (including national decision-makers and local implementation teams) in the design of the implementation strategy and research. Fewer (14 percent, *n* = 68) included measures to strengthen local research capacity, facilitate stakeholder use of findings, or involve stakeholders in the research process.

Assessing whether studies were situated within the realities of implementing organizations and led by local actors was challenging owing to gaps in reporting on funding, management support to interventions, and roles of partners. Approximately 7 percent of studies (*n* = 35) were led by delivery systems or support system actors from the host country and provided detail on how implementation was conducted through routine systems and communities. Forty-five percent (*n* = 212) were either led or supported by international organizations that intervened in implementation conditions and processes; and 48 percent (*n* = 225) of studies did not provide enough detail on implementation arrangements to make a “real world” distinction. Of the thirty-five studies given that classification, ten (29 percent) indicated an intent for the strategy to improve EBP sustainability and two (6 percent) measured this as an implementation outcome.

Finally, we classified studies according to the primary purpose and utility of findings. We found that 38 percent (*n* = 179) of studies primarily addressed whether a strategy helped improve health outcomes, 27 percent (*n* = 128) how to improve health services delivery and management, 19 percent (*n* = 89) how to best design a policy or program, 11 percent (*n* = 52) how to strengthen systems and/or scale up, and 5 percent (*n* = 24) how to empower communities and beneficiaries.

### In-depth review

Of the studies included in full text review, 8 percent (*n* = 38) were eligible for the in-depth review because they met at least five criteria described in Table 5. Twenty-five other studies were included in the in-depth review because they met three criteria and reviewers agreed that they were particularly exemplary vis-à-vis one of them. See Table 5.

#### Focusing on processes and outcomes

Two-thirds of the studies that studied processes and outcomes (*n* = 79) did so to explain the generative process that led to contraceptive uptake. For example, Hackett et al. employed the “implementation outcomes framework” developed by Proctor and colleagues to explain the effectiveness of an intervention to increase postpartum intrauterine device uptake (PPIUD) in the context of a cluster-randomized trial in Tanzania (103). Few studies explained how different implementation variables affect each other along the causal pathway. Abuya et al. examined processes for implementing an SRH voucher intervention in Kenya, explaining how acceptability and feasibility outcomes arise due to specific features of the intervention and how alternative voucher distribution and claim processing practices led to different levels of client satisfaction and efficiency (176). Few studies assessed whether strategies were carried out according to plan. In exception, Karra et al. incorporated implementation fidelity tracking into a cluster-randomized trial to determine if study participants received counseling during pregnancy and estimated an “adherence-adjusted effect” of a PPIUD intervention in Sri Lanka.

Fewer studies that investigated implementation fidelity explored the drivers of patterns. Mayhew et al. retrospectively triangulated an analysis of systems “hardware”, “software” and contextual factors to illuminate barriers and facilitators that influence adherence to SRH-HIV service integration protocols in Kenya (180). Hasselback et al. monitored implementation fidelity associated with an FP supply chain intervention in Senegal and studied whether the program reduced stockouts (181). Overall, there is a paucity of studies that measure whether strategies improve implementation outcomes. A notable exception used organizational network analysis to evaluate whether network strengthening meetings improved the success rate of FP-related referrals between FP and HIV health care providers in Ethiopia (140). Ugaz et al. (2017) found that clinical and business skill trainings to private providers in Nigeria improved the range of FP services available and the quality of counseling (104).

#### Incorporation of contextual analysis

Although one-third of studies reported on use of contextual knowledge or information to adapt implementations strategies, only 42 (27 percent) of them elaborated on an actual contextual analysis. Ayon et al. conducted qualitative formative research to guide efforts to integrate FP-HIV and community outreach strategies for female drug users in Kenya, and Bates et al. did so to inform the design of a private sector capacity building and referral system strengthening intervention in Bangladesh (78, 184). Other studies employed participatory action research (PAR). For example, Tran et al. report on using PAR to adapt postpartum FP interventions in Burkina Faso and the Democratic Republic of the Congo (DRC) and Chhoun et al. to customize mHealth interventions for female entertainment workers in Cambodia (172, 173). EngenderHealth uses the “organizational capacity assessment toolkit”, to tailor capacity building approaches focused on implant and IUD introduction to implementation environments (185). Whereas most studies that use contextual analysis did so to help guide EBP integration into settings, fewer did so to help sustain good practices or scale up (145). Adongo et al. conducted a qualitative appraisal outside Accra, Ghana to inform strategy on adapting the Community-based Health Planning and Systems program, a rural intervention, for urban settlements (186). Projects that used the ExpandNet model incorporated mid-project assessments to guide adaptations of scale up strategies (132–134).

Studies that explained how contextual factors shaped outcomes, for the most part, illuminated the contextual influences on individual reactions or experiences with EBP or implementation strategies, e.g., studies on the contextual conditions that help trigger mechanisms of acceptability of contraception (187, 203–205) and factors that underlie healthcare worker perceptions of the feasibility of adopting EBP or enacting implementation strategies (165, 206, 207). Fewer studies examined organizational adoption of EBP. In exception, the INTEGRA reported on contextual influences that shape healthcare teams' ability to routinely implement integrated SRH-HIV services (68). There is a dearth of contextual analyses that explore scale up and sustainment. Fikree and Zerihun help to fill this gap in a retrospective, mixed method study of factors that helped and hindered a scale up of youth friendly contraceptive services in Ethiopia (120).

#### Research that is fit for purpose and responsive to local demands for knowledge

We observed that few studies described how research designs and methods were adapted to emergent information needs and context. Atchison et al. and Doyle et al. describe the experience of concurrently using human centered design methods (HCD) and designing an evaluation of Adolescent 360 interventions in Ethiopia, Nigeria, and Tanzania (189, 190). The application of HCD involved customizing formative research and testing methods to reflect lessons learned in real time and created opportunities for intervention refinement and optimization. Co-creating the evaluation protocol during these steps enabled researchers to embed data analysis and learning to support adaptive management and course correction during implementation. Hernandez et al. in the DRC describe the experience of the “AcQual” Project in local health zone authorities, which used community-based distributors (CBD) (79). After initial monitoring indicated suboptimal performance of CBD, researchers adapted qualitative and survey research and M&E practices into a multipronged process evaluation that identified where lapses had occurred and informed targeted midcourse corrections that were implemented during the project cycle.

The review identified studies that were responsive to locally articulated demands for evidence on implementation problems. These were, mostly, integration and capacity building strategies to help reposition or improve delivery of the EBP in settings (79, 172); however, there are examples of studies that were embedded in scale ups. Binanga et al. report on embedding IR within policy-decision making and pilot replication processes to help advocate and guide scale up of Sayana Press in the DRC (146). Awoonor-Williams et al. report on similar experiences in Ghana with respect the local to national scale up of community-based primary health care and FP (167).

#### Multisectoral and/or multidisciplinary collaboration

Our review noted studies in which collaboration between stakeholders helped align implementation research, program, policy, and funding cycles. One example of this is an embedded IR project in Burkina Faso led by a consortium of policy decision-makers, WHO, a local research institute, the national FP association, and international NGOs. Together, these actors coordinated a pilot program for task sharing FP services across an integrated cycle that blended demand generation, training, cascaded supervisions, monitoring and evaluation, advocacy, and policy and standard changes (128). Lauria et al. describe a collaborative, multi-sectoral and -disciplinary model used in a pragmatic implementation-effectiveness trial of an integrated community-based health systems strengthening initiative in Togo (192). The study established oversight mechanisms at community, local government and national level and an organizational structure that helps circulate and promote use of input to guide decision-making and adaptations to the implementation strategy.

The review searched for studies of stakeholder collaboration that emphasized evidence utilization and strengthening systemic capacity to adapt and adopt EBP, and found that there was a dearth of research on those subjects. Chitama et al. conducted a mixed-method study in Tanzania to understand the priority setting and planning steps used by district-level healthcare authorities to select and put into practice health EBP including FP (136). The authors recommend revisions to processes of stakeholder engagement, capacity building to strengthen abilities to use EBP, and call for tools to support data use during planning. Three studies focused on country-level stakeholders' adaptation and adoption of WHO global FP guidance and tools. These were noteworthy because of their focus on the strategic and contextual elements that influence whether systems could adapt evidence and put EBP into practice locally, yet the studies were small-scale, and based on qualitative interviews with small numbers of stakeholders (137, 154, 207).

#### Investigating implementation strategies in the “real world”

Studies that we were able to classify as “embedded in the real world” varied in terms of implementation arrangements used. Chabikuli et al. describe an evaluation of the Global HIV/AIDS Initiative Nigeria (GHAIN) FP-HIV integration model, which did not change the basic programmatic and service arrangements within health facilities, but introduced enabling inputs, such as onsite trainings, job aids, and tools to help document and facilitate referral, which were adopted by staff teams that received periodic coaching from GHAIN staff (69). Karim et al. describe an intervention aimed at strengthening Health Extension Program structures in Ethiopia to deliver community-based FP and other health services in which capacity building of frontline staff was carried out by local civil society organizations (194). Other studies evaluated strategies that were led by local delivery systems alone. In Zambia and Mexico, Zulu et al. and Avila-Burgos et al. embedded IR in national policy implementation processes (129, 166). In the former, investigators studied the feasibility and acceptability of using community health assistants to integrate adolescent SRH care into community health systems. Avila-Burgos et al. conducted a longitudinal descriptive analysis of the national Mexican Reproductive Health Subaccounts to determine whether the level and distribution of public revenue for maternal health and FP adhered to the objectives of overarching policies. Phiri et al. and Tweya et al. evaluated FP-HIV care implementation strategies conceived by actors within a small network of clinics in Malawi that operated independently from an external support system actor (195, 196).

Although this review indicates there is a paucity of studies that focused on sustainability, it identified useful examples. In a study on the long-term impact of the Community Health and Family Planning program in northern Ghana, Phillips et al. found that the program effects achieved in the late 1990s-early 2000s were not sustained and associated this with lapses in the delivery of community-driven social and behavioral change communication and male engagement pieces of the original intervention package (161). Speizer et al. examined the sustainability of the Nigerian Urban Reproductive Health Initiative, using longitudinal data on FP service quality and contraceptive attitudes and behaviors between 2011 and 2017 (198, 199). The authors found that although the quality improvements that had been achieved by 2014 had diminished in the subsequent three years, the behavioral effect of implementation in the first phase continued after implementation ended. Fewer studies examined the sustainability of interventions that build organizational capacity. An exception is the PPIUD Initiative in Nepal, Sri Lanka and Tanzania. This study investigated the degree to which the initiative's efforts institutionalized PPIUD training as a regular part of obstetrical-gynecological training programs, measuring whether the personnel trained during the intervention continued to deliver PPIUD counseling and services after the intervention ended and if new personnel were trained in PPIUD by peers that were trained during the intervention (105). Qualitatively, these studies examined how the program affected organizational culture and whether changes supported the spread and sustainment of PPIUD service provision.

## Discussion

Our review illuminated ways in which programs and researchers have harnessed IS to advance FP in LMIC. Though it synthesized the extensive literature that has valuably contributed to these efforts it also identified gaps in the formulation and description of implementation strategies, and inconsistencies in the application of IS theories, frameworks, principles, and research methods. Recognizing these is important so that future IS efforts can generate new knowledge where gaps exist and report findings in a manner that better supports use of new knowledge outside of studies’ original settings.

Most studies examined strategies aimed at improving contraceptive use whereas relatively few prioritized implementation outcomes. This underscores the need for future FP research to adopt a focus on strategies to improve the coverage, implementation, and sustainability EBP in LMIC healthcare landscapes. Studies tended to lack a consistent and thorough delineation of the roles and relationships of stakeholders involved in implementation, and information shared to specify strategies tended to lack appreciable detail on the determinants they targeted, intervention components and how implementing them was to generate the desired outcomes. Future studies should address these gaps since specifying implementation strategies is key to enabling readers to best assess findings and use implementation lessons to inform policies and programs beyond the research environment.

Integration strategies were the most common in this review. Most of these aimed at repositioning FP within the sphere of the user and target factors that impede or enhance the opportunities, intentions, and abilities of the client to use a method. There were a few studies that targeted factors in the environment of implementers to improve the opportunities, intentions, and abilities of health workers to adopt and deliver FP-related EBP and reach populations equitably with those services. Capacity building strategies tended to focus on building individuals' capacity to deliver specific EBP, rather than the capacity of teams and organizations to adopt and sustain, i.e., “institutionalize” EBP implementation, or nuanced approaches to improving upon standard training interventions.

Studies of strategies that sought to spread EBP were relatively few given the recent emphasis placed on scaling up. The review noted the tendency to study scaling up as a special project experience that engaged government programs and local health systems, rather than health system initiatives led by governments that received support implementation partners. This underscores the need for strategies that strengthen how public health systems not only enhance motivation and capacity to integrate specific EBP into routine practice, but also for improving the processes health systems employ to expand effective coverage of EBP generally. There was a paucity of dissemination strategies and among them, all but a few involved social and behavioral communication interventions to affect FP ideation and behaviors. Future projects ought to increase knowledge on dissemination and what works to promote a large scale understanding of FP-related policies and how to implement them. While noting this, it is important to acknowledge that funding constraints, frequently, prevent studies from adopting the extensive scope and timelines required to evaluate scale up and dissemination, both of which take place over long periods and large geographic areas.

Implementation process strategies seek to improve how well teams execute processes to select, adapt and integrate EBP into routine practice. While there is a need to expand the use of such strategies in general, this may especially be the case for strategies that strengthen frameworks and improve processes, already in place in many LMIC health systems, for fostering engagement of stakeholders, and annual planning and monitoring of local health care program implementation. Of the implementation process strategies identified, very few were led by teams that identify, translate, and disseminate knowledge on EBP, i.e., synthesis and translation systems actors. Future projects should focus on ways to expand and optimize the role of these actors to accelerate the spread of EBP adaptation and adoption in countries' national FP programs.

The use of theories, models and frameworks is critical in IS to help studies gain insights into the ways in which implementation strategies are (in)effective. The dearth of studies that explicitly employed these represents a gap since it undermines the ability of researchers to synthesize evidence on implementation outcomes, adapt them to external settings and establish generalizable understandings of the effect of strategies, determinants, and process of translating evidence into practice.

Among the most distinguishing characteristics of IS is the primacy of the implementation outcome variable. Yet, our review found that over a quarter of studies eligible for full-text review did not include them, and of “implementation-effectiveness hybrid” studies, nearly three-fourths prioritize effectiveness measures. FP research stakeholders, including funders, should reflect critically on the degree to which donor interests and the timing and timelines of projects contribute to this gap. In addition, stakeholders should consider lessons from the IS literature which demonstrate the value of measuring implementation outcomes, particularly how doing so provides key insights for implementation. The relative brevity of project timelines precluded most studies from demonstrating the effect of routinely monitoring implementation outcomes, and gleaning lessons from this for adaptation, on downstream health and behavioral effects of programs. Nevertheless, the review provides some illustrative examples of why monitoring implementation outcomes is important: Karim et al. and Spring et al. describe how the application of behavioral economics in formative research generated valuable insights on the acceptability of features of FP interventions, which implementers used to design strategies that resulted in better method continuation and uptake (191, 194). Tawfik et al. describe how the monitoring of implementation fidelity during quality improvement cycles drove iterative improvements in the integration and uptake of postpartum FP (144). Phillips et al., Hernandez et al. and Hasselback et al. leveraged the availability of monitoring data on implementation fidelity to diagnose lapses in management and program delivery processes, identify solutions and inform decisions to improve the health and behavioral effect of FP programs (79, 161, 181).

In highlighting these examples, we call for programs to select implementation outcome variables that are coherent with the underlying theory of change and local, practice needs for implementation evidence, and incorporate more rigorous methods to measuring them during routine monitoring and evaluation. Furthermore, FP funders and others in influential roles should consider the need for extended program and study timelines so that researchers and implementers can do such work, pause and reflect on insights that implementation monitoring produces, and undertake programmatic deliberations with stakeholders accordingly. Researchers are encouraged to apply IS theories and frameworks to this issue and study processes and determinants evidence utilization during programmatic cycles and generate guidance on how to make this effective. To maximize learning, metrics on implementation outcomes and knowledge translation should be linked with effectiveness measures (e.g., FP method uptake) to enable understanding of the dynamics between these three key elements of FP programming.

In addition, regarding measurement and use of implementation outcome variables, we noted few examples of studies that evaluated implementation costs, cost effectiveness and affordability. Similarly, among the reviewed studies we observed that limited scientific attention has been directed at understanding and improving the sustainability of FP programs. We echo the remarks of earlier works that have reflected upon this gap and call for more rigorous economic evaluations and research on the determinants of and strategies to enhance the sustainability of FP interventions (208, 209). Most studies that sought to explain barriers and facilitators elucidated why interventions affected health and behavioral change rather than implementation outcomes. Most examinations of integrating EBP within settings did not use theory to critically examine knowledge translation, and usually gave this information in the way of segueing into reporting on evaluations of effectiveness of EBP introduction on a health outcome.

Few studies evaluated changes in implementation outcomes over time or between study groups. Thus, there is little evidence on strategies for improving EBP coverage, implementation, and sustainability in terms of feasibility, costs, and acceptability. Scant in the reviewed literature were findings on the interplay between implementation outcome variables. This is problematic for evaluations of scaling up, which should explain, for example, whether implementation quality and costs change as coverage increases and over time. In addition, future diffusion and scale up research should fill evidence gaps on the barriers and facilitators to the adoption of FP-related EBP in health systems, strategies that accelerate pace and completeness of scaling up, and factors that influence whether EBP implementation becomes institutionalized where introduced.

Despite the emphasis of IS on evaluating complexity in adaptive systems, the sample of studies we reviewed did not reflect this ideally. Eighteen percent of studies combined quantitative and qualitative research methods and 25 percent evaluated processes and outcomes. Underlying this observation was the lack of consistent reporting vis-à-vis key principles of IS, including whether there were deviations from implementation plans and the degree to which research collaborators positioned their studies within implementation and decision-making systems. Moreover, the studies reviewed provided little information on whether they adapted protocols and methods to ensure contextual relevance and fitness for purpose, the effect of contextual factors on implementation, and the extent to which interventions were applied by and within “real world” implementation systems. In elucidating these gaps, this review calls for more rigorous design and reporting practices to maximize the potential value of IS to FP programs in LMIC. Finally, we observed a dearth of studies which meaningfully included communities and health system stakeholders in evaluation design, including the use of adaptive measures for assessing dynamic interventions and the effects of contextual shifts. In pointing this out, we help illuminate the need for more significant involvement of communities and local level implementers. Specifically, IS practitioners should emphasize steps that garner these actors' ownership of the strategic implementation and research process early, provide frequent opportunities for learning and collaboration between communities and the formal health system, and build local capacity to sustain the benefits that arise applying of IS.

This systematic review has limitations. As described above, the review identified gaps in the specification of interventions and inconsistent reporting on study characteristics. Studies included in the full-text review were not immune to these gaps and yet the review team strove to synthesize their findings, anyway, believing that this would maximize the overall value of the review. This may have resulted in the misclassification of some implementation strategies and overestimation of the prevalence of key descriptors of IS. Studies were deemed eligible for full-text review based on the criteria described above rather than explicit statements of authors. The authors of the reviewed work were not bound to follow specific study design or reporting criteria, which emerged in the IS field during the period of the review with limited cross-over into field of FP research. Therefore, this paper should be received as an examination of how IS has been operationalized and recommendations on ways to strengthen its application, not a critique of authors' works. In addition, journal word limits and writing requirements can restrict what is included in publications. We observed that some projects published numerous studies which have addressed different review criteria, and yet the analysis offered in these pages is of individual studies. Finally, our review only considered studies that were disseminated in English and, therefore, may have missed valuable studies that were written up in other languages.

## Conclusion

As the deadline for achieving SDG approaches, countries' need for contextually appropriate, effective, and sustainable strategies to reach populations with evidence-based FP interventions grows more acute. Our review has demonstrated that IS has promise to support the strengthening FP programs in LMIC, but there is more work to do. We propose three recommendations to maximize its potential to support large-scale impact: (1) donors and Ministries of Health should establish strong structures for research agenda setting that position FP research within implementation and decision-making systems, facilitate multi-stakeholder collaboration, ensure that FP research occurs under usual management conditions, and promote a pragmatic paradigm for FP research that is responsive to context and flexible in studying implementation strategies; (2) FP research should address critical implementation issues such as scale up of complex interventions, the institutional capacity to sustain them, the dissemination of policies and programs, and the translation of knowledge on FP-related EBP into routine implementation and; (3) donors and global FP research leaders should commit to strengthening research capacity in LMIC, including within ministries of health, local health systems and in universities and research institutions, ensuring that FP research, and the benefits that arise from it, unfold in a manner that is equitable and most conducive to evidence use at national levels.

Better reporting of IS is needed so that consumers of FP literature can more thoroughly understand and apply the strategies, concepts and findings associated with efforts to improve FP programs in LMIC. Even though this review identified several strong pieces of research, to enhance learning and the delivery of FP-related EBP more widely, there is a need for more high-quality peer reviewed publications that reflect the full range of implementation problems, contexts, and strategies, and the use of more rigorous and adaptive research methods and designs.

## Data Availability

The data set used for the first stage of the full-text review is available from the authors upon reasonable requests. Requests to access this dataset should be directed to cbaynes@uw.edu.

## Footnotes

^1^EBPs in this paper are defined as programs, practices, principles, procedures, products, pills (or other treatments), and policies that are proven to improve health behaviors or outcomes.

^2^2007 was selected as a boundary for the review because in this year the WHO published the book “Scaling up health service delivery: from pilot innovations to policies and programmes”, which synthesized a great deal of the work which had applied implementation science to advance FP programs previously and directed attention at the need for doing more of this in future. It also established a parameter which allowed the authors to maintain a manageable set of titles/abstracts for review.

## References

[1] USAID. High Impact Practices in Family Planning (HIP). Family planning high impact practices list (2019). Available at: https://www.fphighimpactpractices.org/briefs/family-planning-high-impact-practices-list/ (Accessed on December 1, 2021).

[2] Rusatira JC Silberg C Mickler A Salmeron C Twahirwa Rwema JO Johnstone M Family planning science and practice lessons from the 2018 international conference on family planning. Gates Open Res. (2021) 4:4–43. 10.12688/gatesopenres.13130.2 PMC7374012 32760880

[3] Mwaikambo L Speizer IS Schurmann A Morgan G Fikree F. What works in family planning interventions: a systematic review. Stud Fam Plann. (2011) 42(2):67–82. 10.1111/j.1728-4465.2011.00267.x 21834409 PMC3761067

[4] Thatte N Cuzin-Kihl A May AV D’Adamo M Addico G Kiarie J Leveraging a partnership to disseminate and implement what works in family planning and reproductive health: the implementing best practices (IBP) initiative. Glob Health Sci Pract. (2019) 7(1):12–9. 10.9745/GHSP-D-18-00236 30926735 PMC6538130

[5] Subramanian S Naimoli J Matsubayashi T Peters DH. Do we have the right models for scaling up health services to achieve the millennium development goals? BMC Health Serv Res. (2011) 11:336. 10.1186/1472-6963-11-336 22168915 PMC3260120

[6] Hardee K Suzbach S Chatterji M Reier S Malarcher S. Guidance on assessing the potential sustainability of practices as part of an evidence review: considerations for high impact practices in family planning. Washington, D.C: United States Agency for International Development (2017).

[7] Simmons R Brown J Diaz M. Facilitating large-scale transitions to quality of care: an idea whose time has Come. Stud Fam Plann. (2002) 33(1):61–75. 10.1111/j.1728-4465.2002.00061.x 11974420

[8] Brown CH Curran G Palinkas LA Aarons GA Wells KB Jones L An overview of research and evaluation designs for dissemination and implementation. Annu Rev Public Health. (2017) 38(1):1–22. 10.1146/annurev-publhealth-031816-044215 28384085 PMC5384265

[9] Eccles MP Mittman BS. Welcome to implementation science. Implement Sci. (2006) 1(1):1–3. 10.1186/1748-5908-1-1

[10] Phillips JF MacLeod BB Kachur SP. Bugs in the bed: addressing the contradictions of embedded science with Agile implementation research. Glob Health Sci Pract. (2021) 9(1):55–77. 10.9745/GHSP-D-20-00169 33795362 PMC8087429

[11] Ghiron L Ramirez-Ferrero E Badiani R Benevides R Ntabona A Fajans P Promoting scale-up across a global project platform: lessons from the evidence to action project. Glob Implement Res Appl. (2021) 1(2):69–76. 10.1007/s43477-021-00013-4

[12] Binagwaho A Frisch MF Udoh K Drown L Ntawukuriryayo JT Nkurunziza D Implementation research: an efficient and effective tool to accelerate universal health coverage. Int J Health Policy Manag. (2020) 9(5):182–184. 10.1186/s12913-021-06449-6 32563218 PMC7306110

[13] Nnaji CA Wiysonge CS Okeibunor JC Malinga T Adamu AA Tumusiime P Implementation research approaches to promoting universal health coverage in Africa: a scoping review. BMC Health Serv Res. (2021) 21:414. 10.1186/s12913-021-06449-6 33941178 PMC8094606

[14] Peters DH. Health policy and systems research: the future of the field. Health Res Policy Syst. (2018) 16:84. 10.1186/s12961-018-0359-0 30134979 PMC6103853

[15] Peterson HB Haidar J Fixsen D Ramaswamy R Weiner BJ Leatherman S. Implementing innovations in global Women's, Children's, and Adolescents’ health. Obstet Gynecol. (2018) 131(3):423–430. 10.1097/AOG.0000000000002494 29420393

[16] Hardee K Wright K Spicehandler J. Family planning policy, program, and practice decisionmaking: the role of research evidence and other factors. Washington, D.C: Population Council (2015).

[17] Peters DH Adam T Alonge O Agyepong IA Tran N. Republished research: implementation research: what it is and how to do it. Br J Sports Med. (2014) 48(8):731–6. 10.1136/bmj.f6753 24659611

[18] Foreit J Frejka T. Family planning operations research: a book of readings. New York, NY: The Population Council (1998).

[19] Wawer MJ McNamara R McGinn T Lauro D. Family planning operations research in Africa: reviewing a decade of experience. Stud Fam Plann (1991) 22(5):279–293. 10.2307/1966683 1759274

[20] Phillips JF Simmons R Simmons GB Yunus M. Transferring health and family planning service innovations to the public sector : an experiment in organization development in Bangladesh. Stud Fam Plann. (1984) 15(2):62–73. 10.2307/1966047 6710550

[21] Entwisle B. Measuring components of family planning program effort. Demography. (1989) 26(1):53–76. 10.2307/2061493 2737358

[22] Mauldin WP Lapham RJ. The measurement of family planning inputs. In: Lapham RJ Simmons B, editors. Organizing for effective family planning programs. Washington, D.C: National Academy Press (1987). p. 545–82.

[23] Simmons R Hall P Díaz J Díaz M Fajans P Satia J. The strategic approach to contraceptive introduction. Stud Fam Plann. (1997) 28(2):79–94. 10.2307/2138111 9216029

[24] Fajans P Simmons R Ghiron L. Helping public sector health systems innovate: the strategic approach to strengthening reproductive health policies and programs. Am J Public Health. (2006) 96(3):435–40. 10.2105/AJPH.2004.059907 16449594 PMC1470499

[25] Measham D Haberland N. Responding to Cairo: case studies of changing practice in reproductive health and family planning. 1st ed. New York, NY: The Population Council (2002).

[26] McIntosh CA Finkle JL. The Cairo conference on population and development: a new paradigm? Popul Dev Rev. (1995) 21(2):223–260. 10.2307/2137493

[27] Wilcher R Cates W Gregson S. Family planning and HIV: strange bedfellows no longer. AIDS. (2009) 23(Suppl 1):S1–6. 10.1097/01.aids.0000363772.45635.35 20081381 PMC3516801

[28] Ross J Stover J Adelaja D. Family planning programs in 2004: new assessments in a changing environment. Int Fam Plan Perspect. (2007) 33(1):22–30. 10.1363/3302207 17462985

[29] Crossette B. Reproductive health and the millennium development goals: the missing link. Stud Fam Plann. (2005) 36(1):71–79. 10.1111/j.1728-4465.2005.00042.x 15828526

[30] Simmons R Shiffman JS. Scaling up health service innovations: a framework for action. In: Simmons R Fajans P Ghiron L, editors. Scaling up health service delivery : from pilot innovations to policies and programmes. Geneva: World Health Organisation (2007). p. 1–31.

[31] Theobald S Brandes N Gyapong M El-Saharty S Proctor E Diaz T Implementation research: new imperatives and opportunities in global health. Lancet. (2018) 392(10160):2214–28. 10.1016/S0140-6736(18)32205-0 30314860

[32] Pinnock H Barwick M Carpenter CR Eldridge S Grandes G Griffiths CJ Standards for reporting implementation studies (StaRI) statement. Br Med J. (2017) 356:1–9. 10.1136/bmj.i6795 PMC5421438 28264797

[33] World Health Organization, USAID, World Bank Group, University Research CoL. Statement on advancing implementation research and delivery science. Cape Town, South Africa: Alliance for Health Policy and Systems Research (2014) Available at: https://healthsystemsglobal.org/wp-content/uploads/2020/05/statement_IRDS.pdf (Accessed January 6, 2022).

[34] Proctor EK Powell BJ McMillen JC. Implementation strategies: recommendations for specifying and reporting. Implement Sci. (2013) 8(1):1–11. 10.1186/1748-5908-8-1 24289295 PMC3882890

[35] Leeman J Birken SA Powell BJ Rohweder C Shea CM. Beyond “implementation strategies”: classifying the full range of strategies used in implementation science and practice. Implement Sci. (2017) 12(1):1–9. 10.1186/s13012-017-0657-x 29100551 PMC5670723

[36] Powell BJ Waltz TJ Chinman MJ Damschroder LJ Smith JL Matthieu MM A refined compilation of implementation strategies: results from the expert recommendations for implementing change (ERIC) project. Implement Sci. (2015) 10(1):1–14. 10.1186/s13012-015-0209-1 25889199 PMC4328074

[37] Kazdin AE. Evidence-based treatment and practice: new opportunities to bridge clinical research and practice, enhance the knowledge base, and improve patient care. American Psychologist. (2008) 63(3):146–59. 10.1037/0003-066X.63.3.146 18377105

[38] Grimshaw JM Eccles MP Lavis JN Hill SJ Squires JE. Knowledge translation of research findings. Implement Sci. (2012) 7(1):50. 10.1186/1748-5908-7-50 22651257 PMC3462671

[39] Bosch M van der Weijden T Wensing M Grol R. Tailoring quality improvement interventions to identified barriers: a multiple case analysis. J Eval Clin Pract. (2007) 13(2):161–168. 10.1111/j.1365-2753.2006.00660.x 17378860

[40] Fixsen DL Naoom SF Blase KL Friedman RM Wallace F. Implementation Research: A Synthesis of the Literature. Report No.: 231. Tampa, FL. (2005).

[41] Lobb R Colditz GA. Implementation science and its application to population health. Annu Rev Public Health. (2013) 34:235–51. 10.1146/annurev-publhealth-031912-114444 23297655 PMC3901430

[42] Bartels SM Haider S Williams CR Mazumder Y Ibisomi L Alonge O Diversifying implementation science: a global perspective. Glob Health Sci Pract. (2022) 10(4):e2100757. 10.9745/GHSP-D-21-00757 36041849 PMC9426981

[43] Palinkas LA Aarons GA Horwitz S Chamberlain P Hurlburt M Landsverk J. Mixed method designs in implementation research. Adm Policy Ment Health. (2011) 38(1):44–53. 10.1007/s10488-010-0314-z 20967495 PMC3025112

[44] Curran GM Mittman B Pyne JM Stetler C Bauer M. Effectiveness-implementation hybrid designs: combining elements of clinical effectiveness and implementation research to enhance public health impact. Med Care. (2012) 50(3):217–26. 10.1097/MLR.0b013e3182408812 22310560 PMC3731143

[45] Edwards A Zweigenthal V Olivier J. Evidence map of knowledge translation strategies, outcomes, facilitators and barriers in african health systems. Health Res Policy Syst. (2019) 17:16. 10.1186/s12961-019-0419-0 30732634 PMC6367796

[46] Menear M Blanchette MA Demers-Payette O Roy D. A framework for value-creating learning health systems. Health Res Policy Syst. (2019) 17(1):79. 10.1186/s12961-019-0477-3 31399114 PMC6688264

[47] Alliance for Health Policy and Systems Research. (2018). Available at: https://www.who.int/alliance-hpsr/resources/Alliance-embedded-hpsr-BriefingNote-WEB.pdf (Accessed on January 6, 2022).

[48] Moher D Liberati A Tetzlaff J Altman DG. Preferred reporting items for systematic reviews and meta-analyses: the PRISMA statement. PLoS Med. (2009) 6(7):e1000097. 10.1371/journal.pmed.1000097 19621072 PMC2707599

[49] Effective Public Healthcare Panacea Project. Quality assessment tool for quantitative studies (2012). Available at: https://www.ephpp.ca/quality-assessment-tool-for-quantitative-studies/ (Accessed on June 18, 2021).

[50] CASP (Critical Appraisal Skills Program). Casp checklist: 10 questions to help you make sense of a qualitative research (2018). Available at: https://casp-uk.net/wp-content/uploads/2018/03/CASP-Qualitative-Checklist-2018_fillable_form.pdf (Accessed on June 18, 2021).

[51] World Health Organization. Everybody's business: strengthening health systems to improve health outcomes: wHO's Framework for action. Geneva, Switzerland. (2007).

[52] Mazza D Bairstow P Buchan H Chakraborty SP van Hecke O Grech C Refining a taxonomy for guideline implementation: results of an exercise in abstract classification. Implement Sci. (2013) 8(1):32. 10.1186/1748-5908-8-32 23497520 PMC3606141

[53] Wandersman A Duffy J Flaspohler P Noonan R Lubell K Stillman L Bridging the gap between prevention research and practice: the interactive systems framework for dissemination and implementation. Am J Community Psychol. (2008) 41(3–4):171–81. 10.1007/s10464-008-9174-z 18302018

[54] Damschroder LJ Aron DC Keith RE Kirsh SR Alexander JA Lowery JC. Fostering implementation of health services research findings into practice: a consolidated framework for advancing implementation science. Implement Sci. (2009) 50(4):1–15. 10.1186/1748-5908-4-50 PMC2736161 19664226

[55] Flottorp SA Oxman AD Krause J Musila NR Wensing M Godycki-cwirko M A checklist for identifying determinants of practice : a systematic review and synthesis of frameworks and taxonomies of factors that prevent or enable improvements in healthcare professional practice. Implement Sci. (2013) 8:35. 10.1186/1748-5908-8-35 23522377 PMC3617095

[56] Proctor E Silmere H Raghavan R Hovmand P Aarons G Bunger A Outcomes for implementation research: conceptual distinctions, measurement challenges, and research agenda. Adm Policy Ment Health. (2011) 38(2):65–76. 10.1007/s10488-010-0319-7 20957426 PMC3068522

[57] Leeman J Calancie L Hartman MA Escoffery CT Herrmann AK Tague LE What strategies are used to build practitioners’ capacity to implement community-based interventions and are they effective?: a systematic review. Implement Sci. (2015) 10:1. 10.1186/s13012-015-0272-7 26018220 PMC4449971

[58] Meyers DC Durlak JA Wandersman A. The quality implementation framework: a synthesis of critical steps in the implementation process. Am J Community Psychol. (2012) 50(3–4):462–80. 10.1007/s10464-012-9522-x 22644083

[59] McCormack L Sheridan S Lewis M Boudewyns V Melvin KL Kistler C Communication and dissemination strategies to facilitate the use of health-related evidence. Rockville, MD, USA: Agency for Healthcare Research and Quality. Evidence Report/Technology Assessment (2013). 10.23970/ahrqepcerta213 PMC4781094 24423078

[60] McCarthy O Ahamed I Kulaeva F Tokhirov R Saibov S Vandewiele M A randomized controlled trial of an intervention delivered by mobile phone app instant messaging to increase the acceptability of effective contraception among young people in Tajikistan. Reprod Health. (2018) 15(1):28. 10.1186/s12978-018-0473-z 29433506 PMC5809875

[61] McCarthy OL Zghayyer H Stavridis A Adada S Ahamed I Leurent B A randomized controlled trial of an intervention delivered by mobile phone text message to increase the acceptability of effective contraception among young women in palestine. Trials. (2019) 20(1):228. 10.1186/s13063-019-3297-4 31014358 PMC6477750

[62] Smith C Ly S Uk V Warnock R Edwards P Free C. Process evaluation of a mobile phone-based intervention to support post-abortion contraception in Cambodia. Contracept Reprod Med. (2017) 2(1):16. 10.1186/s40834-017-0043-8 29201421 PMC5683466

[63] Harrington EK Drake AL Matemo D Ronen K Osoti AO John-Stewart G An mHealth SMS intervention on postpartum contraceptive use among women and couples in Kenya: a randomized controlled trial. Am J Public Health. (2019) 109:934–941. 10.2105/AJPH.2019.305051 31067089 PMC6507993

[64] McConnell M Rothschild CW Ettenger A Muigai F Cohen J. Free contraception and behavioural nudges in the postpartum period: evidence from a randomised control trial in Nairobi, Kenya. BMJ Glob Health. (2018) 3(5):e000888. 10.1136/bmjgh-2018-000888 30364345 PMC6195134

[65] Bates LA Hicks JP Walley J Robinson E. Evaluating the impact of marie stopes international's digital family planning counselling application on the uptake of long-acting and permanent methods of contraception in Vietnam and Ethiopia: a study protocol for a multi-country cluster randomised controlled trial. Trials. (2018) 19:420. 10.1186/s13063-018-2815-0 30075739 PMC6091072

[66] Reiss K Andersen K Barnard S Ngo TD Biswas K Smith C Using automated voice messages linked to telephone counselling to increase post-menstrual regulation contraceptive uptake and continuation in Bangladesh: study protocol for a randomised controlled trial. BMC Public Health. (2017) 17(1):769. 10.1186/s12889-017-4703-z 28974209 PMC5627401

[67] Reiss K Andersen K Pearson E Biswas K Taleb F Ngo TD Unintended consequences of mHealth interactive voice messages promoting contraceptive use after menstrual regulation in Bangladesh: intimate partner violence results from a randomized controlled trial. Glob Health Sci Pract. (2019) 7(3):386–403. 10.9745/GHSP-D-19-00015 31558596 PMC6816818

[68] Church K Wringe A Lewin S Ploubidis GB. Exploring the feasibility of service integration in a low-income setting : a mixed methods investigation into different models of reproductive health and HIV care in. PLoS One. (2015) 10(5):1–19. 10.1371/journal.pone.0126144 PMC4433110 25978632

[69] Chabikuli NO Awi DD Chukwujekwu O Abubakar Z Gwarzo U Ibrahim M The use of routine monitoring and evaluation systems to assess a referral model of family planning and HIV service integration in Nigeria. AIDS. (2009) 23:S1–S103. 10.1097/01.aids.0000363782.50580.d8 20081394

[70] Dulli L Field S Masaba R Ndiritu J. Addressing broader reproductive health needs of female sex workers through integrated family planning/ HIV prevention services: a non-randomized trial of a health-services intervention designed to improve uptake of family planning services in Kenya. PLoS One. (2019) 14(7):e0219813. 10.1371/journal.pone.0219813 31339919 PMC6655688

[71] Tran NT Gaffield ME Seuc S Landoulsi S Yameogo WME Cuzin-Kihl A Effectiveness of a package of postpartum family planning interventions on the uptake of contraceptive methods until twelve months postpartum in Burkina Faso and the democratic Republic of Congo: the YAM DAABO study protocol. BMC Health Serv Res (2018) 18:439. 10.1186/s12913-018-3199-2 29890982 PMC5996535

[72] Tran NT Yameogo WME Gaffield ME Langwana F Kiarie J Kulimba DM Postpartum family-planning barriers and catalysts in Burkina Faso and the democratic Republic of Congo: a multiperspective study. Open Access J Contracept. (2018) 9:63–74. 10.2147/OAJC.S170150 30519124 PMC6236096

[73] Warren CE Mayhew SH Vassall A Kimani JK Church K Obure CD Study protocol for the Integra initiative to assess the benefits and costs of integrating sexual and reproductive health and HIV services in Kenya and Swaziland. BMC Public Health. (2012) 12(1):973. 10.1186/1471-2458-12-973 23148456 PMC3529107

[74] Ojanduru L Ojamuge D DuComb L Cachan J Spindler E. Testing a proof of concept model for group couples counseling in family planning in northern Uganda. Pan Afr Med J. (2018) 30:179. 10.11604/pamj.2018.30.179.12670 30455808 PMC6235475

[75] Biswas KK Pearson E Shahidullah SM Sultana S Chowdhury R Andersen KL. Integrating postabortion care, menstrual regulation and family planning services in Bangladesh: a pre-post evaluation. Reprod Health. (2017) 14(1):37. 10.1186/s12978-017-0298-1 28284230 PMC5346262

[76] Grossman D Onono M Newmann SJ Blat C Bukusi E Shade SB. Integration of family planning services into HIV care and treatment in Kenya: a cluster-randomized trial. AIDS. (2013) 27(Suppl 1):S77–85. 10.1097/QAD.0000000000000035 24088687

[77] Farrokh-Eslamlou H Aghlmand S Eslami M Homer CSE. Impact of the world health organization's decision-making tool for family planning clients and providers on the quality of family planning services in Iran. J Fam Plann Reprod Health Care. (2014) 40(2):89–95. 10.1136/jfprhc-2012-100290 23946327

[78] Bates L Huque R Bhowmik P King R Elsey H Newell J Partnering with private providers to promote long-acting contraceptives in urban Bangladesh: a mixed-methods feasibility study. Int Perspect Sex Reprod Health. (2019) 45:87–99. 10.1363/45e8219 31895041

[79] Hernandez JH Akilimali PZ Muanda MF Glover AL Bertrand JT. Evolution of a large-scale community-based contraceptive distribution program in Kinshasa, DRC based on process evaluation. Glob Health Sci Pract. (2018) 6(4):657–67. 10.9745/GHSP-D-18-00205 30591574 PMC6370360

[80] Baqui AH Ahmed S Begum N Khanam R Mohan D Harrison M Impact of integrating a postpartum family planning program into a community-based maternal and newborn health program on birth spacing and preterm birth in rural Bangladesh. J Glob Health. (2018) 8(2):20406. 10.7189/jogh.08.020406 PMC6036944 30023053

[81] Mudiope P Musingye E Makumbi CO Bagenda D Homsy J Nakitende M Greater involvement of HIV-infected peer-mothers in provision of reproductive health services as “family planning champions” increases referrals and uptake of family planning among HIV-infected mothers. BMC Health Serv Res. (2017) 17(1):444. 10.1186/s12913-017-2386-x 28655314 PMC5488413

[82] Cover J Namagembe A Tumusiime J Nsangi D Lim J Nakiganda-busiku D. Continuation of injectable contraception when self-injected vs. Administered by a facility-based health worker : a nonrandomized, prospective cohort study in Uganda. Contraception. (2018) 98(5):383–8. 10.1016/j.contraception.2018.03.032 29654751 PMC6197833

[83] Burke HM Packer C Buluzi M Healy E Ngwira B. Client and provider experiences with self-administration of subcutaneous depot medroxyprogesterone acetate (DMPA-SC) in Malawi. Contraception. (2018) 98(5):405–410. 10.1016/j.contraception.2018.02.011 29706227

[84] Burke HM Chen M Buluzi M Fuchs R Wevill S Venkatasubramanian L Women's satisfaction, use, storage and disposal of subcutaneous depot medroxyprogesterone acetate (DMPA-SC) during a randomized trial. Contraception. (2018) 98(5):418–22. 10.1016/j.contraception.2018.04.018 29758176

[85] Burke HM Chen M Buluzi M Fuchs R Wevill S Venkatasubramanian L Articles effect of self-administration versus provider-administered injection of subcutaneous depot medroxyprogesterone acetate on continuation rates in Malawi : a randomised controlled trial. Lancet Glob Health. (2018) 6(5):e568–78. 10.1016/S2214-109X(18)30061-5 29526707

[86] Mullany LC Lee CI Yone L Paw P Oo EKS Maung C Access to essential maternal health interventions and human rights violations among vulnerable communities in eastern Burma. PLoS Med. (2008) 5(12):1689–98. 10.1371/journal.pmed.0050242 19108601 PMC2605890

[87] Huda FA Mahmood HR Ahmmed F Ahmed A Hassan AT Panza A The effect of a club in making differences in knowledge, attitude, and practices on family planning among married adolescent girls in urban slums in Bangladesh. Int J Environ Res Public Health. (2019) 16(20):4037. 10.3390/ijerph16204037 31652488 PMC6844075

[88] Bapolisi WA Ferrari G Blampain C Makelele J Bisimwa G Merten S. Impact of a complex gender-transformative intervention on maternal and child health outcomes in the eastern democratic Republic of Congo: protocol of a longitudinal parallel mixed-methods study. BMC Public Health. (2020) 20(1):51. 10.1186/s12889-019-8084-3 31937267 PMC6961329

[89] Challa S DeLong SM Carter N Johns N Shakya H Boyce SC Protocol for cluster randomized evaluation of reaching married adolescents-a gender-synchronized intervention to increase modern contraceptive use among married adolescent girls and young women and their husbands in Niger. Reprod Health. (2019) 16(1):180. 10.1186/s12978-019-0841-3 31852538 PMC6921454

[90] Chirwa-Kambole E Svanemyr J Sandøy I Hangoma P Zulu JM. Acceptability of youth clubs focusing on comprehensive sexual and reproductive health education in rural Zambian schools: a case of central province. BMC Health Serv Res. (2020) 20(1):42. 10.1186/s12913-020-4889-0 31948452 PMC6966797

[91] Subramanian L Simon C Daniel EE. Increasing contraceptive use among young married couples in bihar, India: evidence from a decade of implementation of the PRACHAR project. Glob Health Sci Pract. (2018) 6(2):330–44. 10.9745/GHSP-D-17-00440 29959273 PMC6024625

[92] Oyugi B Kioko U Kaboro SM Gikonyo S Okumu C Ogola-Munene S Accessibility of long-term family planning methods: a comparison study between output based approach (OBA) clients verses non-OBA clients in the voucher supported facilities in Kenya. BMC Health Serv Res. (2017) 17(1):236. 10.1186/s12913-017-2164-9 28347306 PMC5368892

[93] Ali M Azmat SK Hamza HB Rahman MM Hameed W. Are family planning vouchers effective in increasing use, improving equity and reaching the underserved? An evaluation of a voucher program in Pakistan. BMC Health Serv Res. (2019) 19(1):200. 10.1186/s12913-019-4027-z 30922318 PMC6440079

[94] Atukunda EC Mugyenyi GR Obua C Atuhumuza EB Lukyamuzi EJ Kaida A Provision of family planning vouchers and early initiation of postpartum contraceptive use among women living with HIV in southwestern Uganda: a randomized controlled trial. Todd CS, editor. PLoS Med. (2019) 16(6):e1002832. 10.1371/journal.pmed.1002832 31226123 PMC6588214

[95] Bellows B Warren C Vonthanak S Chhorvann C Sokhom H Men C Evaluation of the impact of the voucher and accreditation approach on improving reproductive behaviors and status in Cambodia. BMC Public Health. (2011) 11(1):667. 10.1186/1471-2458-11-667 21864405 PMC3170624

[96] Reiss K Penfold S Alabi O Ali M Hopkins K Dinh Ngo T Safety, quality, and acceptability of contraceptive subdermal implant provision by community health extension workers versus nurses and midwives in Nigeria: protocol for a quasi-experimental, noninferiority study. J Med Internet Res. (2018) 7(3):e67. 10.2196/resprot.8721 PMC5856922 29500162

[97] Jacinto A Mobaracaly MR Ustáb MB Bique C Blazer C Weidert K Safety and acceptability of community-based distribution of injectable contraceptives: a pilot project in Mozambique. Glob Health Sci Pract. (2016) 4(3):410–21. 10.9745/GHSP-D-16-00133 27651076 PMC5042697

[98] Cover L Namagembe T Drake C. Acceptability of contraceptive self-injection with DMPA-SC among adolescents in Gulu district, Uganda. Int Perspect Sex Reprod Health. (2017) 43(4):153. 10.1363/43e5117 29771679

[99] Cover J Ba M Lim J Drake JK Daff BM. Evaluating the feasibility and acceptability of self-injection of subcutaneous depot medroxyprogesterone acetate (DMPA) in Senegal : a prospective cohort study. Contraception. (2017) 96(3):203–10. 10.1016/j.contraception.2017.06.010 28673645 PMC6381449

[100] di Giorgio L Mvundura M Tumusiime J Morozoff C Cover J Kidwell J. Is contraceptive self-injection cost-effective compared to contraceptive injections from facility-based health workers ? evidence from Uganda. Contraception. (2018) 98(5):396–404. 10.1016/j.contraception.2018.07.137 30098940 PMC6197841

[101] Nuccio O Sendek B Park MH Mesele T Okello FO Gordon-Maclean C. Optimizing tubal ligation service delivery: a prospective cohort study to measure the task-sharing experience of marie stopes international Ethiopia. Health Policy Plan. (2017) 32(2):163–9. 10.1093/heapol/czw105 28207063

[102] Ogu R Okonofua F Hammed A Okpokunu E Mairiga A Bako A Outcome of an intervention to improve the quality of private sector provision of postabortion care in northern Nigeria. Int J Gynecol Obstet. (2012) 118(Suppl. 2):S121–6. 10.1016/S0020-7292(12)60010-1 22920615

[103] Hackett K Huber-Krum S Francis JM Senderowicz L Pearson E Siril H Evaluating the implementation of an intervention to improve postpartum contraception in Tanzania: a qualitative study of provider and client perspectives. Glob Health Sci Pract. (2020) 8(2):270–89. 10.9745/GHSP-D-19-00365 32606094 PMC7326523

[104] Ugaz J Leegwater A Chatterji M Johnson D Baruwa S Toriola M Impact of family planning and business trainings on private-sector health care providers in Nigeria. Int Perspect Sex Reprod Health. (2017) 43(2):51–65. 10.1363/43e3717 29261503

[105] Canning D Shah IH Pearson E Pradhan E Karra M Senderowicz L Institutionalizing postpartum intrauterine device (IUD) services in Sri Lanka, Tanzania, and Nepal: study protocol for a cluster-randomized stepped-wedge trial. BMC Pregnancy Childbirth. (2016) 16(1):362. 10.1186/s12884-016-1160-0 27871269 PMC5117577

[106] Mugore S Kassouta TK Sebikali B Lundstrom L. Improving the quality of postabortion care services in Togo increased uptake of contraception. Glob Health Sci Pract. (2016) 4(3):495–505. 10.9745/GHSP-D-16-00212 27688719 PMC5042703

[107] Karra M Pearson E Pradhan E de Silva R Samarasekera A Canning D The effect of a postpartum IUD intervention on counseling and choice: evidence from a cluster-randomized stepped-wedge trial in Sri Lanka. Trials. (2019) 20(1):407. 10.1186/s13063-019-3473-6 31287021 PMC6615190

[108] Kiemtoré S Zoungrana Z Zamané H Kaboré CWPD Ouédraogo A Bonané B. Interventions to improve the use of long-acting reversible contraceptive methods at primary health centers in Burkina Faso. Int J Gynecol Obstet. (2019) 147(3):350–355. 10.1002/ijgo.12973 31523811

[109] LeFevre A Mpembeni R Kilewo C Yang A An S Mohan D Program assessment of efforts to improve the quality of postpartum counselling in health centers in Morogoro region, Tanzania. BMC Pregnancy Childbirth. (2018) 18(1):282. 10.1186/s12884-018-1906-y 29973185 PMC6031177

[110] Cooper CM Wille J Shire S Makoko S Tsega A Schuster A Integrated family planning and immunization service delivery at health facility and community sites in dowa and ntchisi districts of Malawi: a mixed methods process evaluation. Int J Environ Res Public Health. (2020) 17(12):4530. 10.3390/ijerph17124530 32599688 PMC7345913

[111] Hardee K Jurczynska K Sinai I Boydell V Muhwezi DK Gray K Improving voluntary, rights-based family planning: experience from Nigeria and Uganda. Open Access J Contracept. (2019) 10:55–67. 10.2147/OAJC.S215945 31807091 PMC6839576

[112] Wendot S Scott RH Nafula I Theuri I Ikiugu E Footman K. Evaluating the impact of a quality management intervention on post-abortion contraceptive uptake in private sector clinics in western Kenya: a pre- and post-intervention study. Reprod Health. (2018) 15(1):10. 10.1186/s12978-018-0452-4 29351797 PMC5775589

[113] Zafar Z Habib H Kols A Assad F Lu ER Schuster A. Reinvigorating postpartum intrauterine contraceptive device use in Pakistan: an observational assessment of competency-based training of health providers using low-cost simulation models. BMC Med Educ. (2019) 19:261. 10.1186/s12909-019-1683-y 31307460 PMC6631998

[114] Ndwiga C Birungi H Undie CC Weyenga H Sitienei J. Feasibility and effect of integrating tuberculosis screening and detection in postnatal care services: an operations research study. BMC Health Serv Res. (2013) 13:99. 10.1186/1472-6963-13-99 23496997 PMC3602180

[115] Limaye R J Ballard Sara A Ahmed N Ohkbuo S Deka S Mickish Gross C Enhancing the knowledge and behaviors of fieldworkers to promote family planning and maternal, newborn, and child health in Bangladesh through a digital health training package: results from a pilot study. Int Q Community Health Educ. (2020) 40(2):143–149. 10.1177/0272684X19861866 31274369

[116] Zeng W Shepard DS Nguyen H Chansa C Das AK Qamruddin J Cost–effectiveness of results-based financing, Zambia: a cluster randomized trial. Bull World Health Organ. (2018) 96(11):760–71. 10.2471/BLT.17.207100 30455531 PMC6239017

[117] Zeng W Shepard DS Rusatira JdD Blaakman AP Nsitou BM. Evaluation of results-based financing in the republic of the Congo: a comparison group pre-post study. Health Policy Plan. (2018) 33(3):392–400. 10.1093/heapol/czx195 29351604

[118] Nahimana E McBain R Manzi A Iyer H Uwingabiye A Gupta N Race to the top: evaluation of a novel performancebased financing initiative to promote healthcare delivery in rural Rwanda. Glob Health Action. (2016) 9(1):1–8. 10.3402/gha.v9.32943 27900933 PMC5129093

[119] Friedman J Qamruddin J Chansa C Das AK. Impact evaluation of Zambia's Health results-based financing pilot project. Washington, DC: World Bank Group (2016).

[120] Fikree FF Zerihun H. Scaling up a strengthened youth-friendly service delivery model to include long-acting reversible contraceptives in Ethiopia: a mixed methods retrospective assessment. Int J Health Policy Manag. (2020) 9(2):53–64. 10.15171/ijhpm.2019.76 32124589 PMC7054650

[121] Samuel M Fetters T Desta D. Strengthening postabortion family planning services in Ethiopia: expanding contraceptive choice and improving access to long-acting reversible contraception. Glob Health Sci Pract. (2016) 4(Suppl 2):S60–72. 10.9745/GHSP-D-15-00301 27540126 PMC4990163

[122] Dunbar EL Wroe EB Nhlema B Kachimanga C Gupta R Taylor C Evaluating the impact of a community health worker programme on non-communicable disease, malnutrition, tuberculosis, family planning and antenatal care in neno, Malawi: protocol for a stepped-wedge, cluster randomised controlled trial. BMJ Open. (2018) 8(7):e019473. 10.1136/bmjopen-2017-019473 30007924 PMC6089278

[123] Prata N Downing J Bell S Weidert K. Cost of providing injectable contraceptives through a community-based social marketing program in tigray, Ethiopia. Contraception. (2016) 93(6):485–91. 10.1016/j.contraception.2016.01.017 26872718

[124] Bellows B Mackay A Dingle A Tuyiragize R Nnyombi W Dasgupta A. Increasing contraceptive access for hard-to-reach populations with vouchers and social franchising in Uganda. Glob Health Sci Pract. (2017) 5(3):446–55. 10.9745/GHSP-D-17-00065 28963175 PMC5620340

[125] Pereira SK Kumar P Dutt V Haldar K Penn-Kekana L Santos A Protocol for the evaluation of a social franchising model to improve maternal health in uttar pradesh, India. Implement Sci. (2015) 10(1):1–14. 10.1186/s13012-015-0269-2 26008202 PMC4448271

[126] Liu J Schatzkin E Omoluabi E Fajemisin M Onuoha C Erinfolami T Introducing the subcutaneous depot medroxyprogesterone acetate injectable contraceptive via social marketing: lessons learned from Nigeria's Private sector. Contraception. (2018) 98(5):438–48. 10.1016/j.contraception.2018.07.005 30071196 PMC6197840

[127] Azmat SK Shaikh BT Hameed W Mustafa G Hussain W Asghar J Impact of social franchising on contraceptive use when complemented by vouchers: a quasi-experimental study in rural Pakistan. PLoS One. (2013) 8(9):e74260–e74260. 10.1371/journal.pone.0074260 24069287 PMC3772094

[128] Millogo T Kouanda S Tran NT Kaboré B Keita N Ouedraogo L Task sharing for family planning services, Burkina Faso. Bull World Health Organ. (2019) 97(11):783–8. 10.2471/BLT.19.230276 31673194 PMC6802696

[129] Avila-Burgos L Cahuana-Hurtado L Montanez-Hernandez J Servan-Mori E Aracena-Genao B del Rio-Zolezzi A. Financing maternal health and family planning: are we on the right track? Evidence from the reproductive health subaccounts in Mexico, 2003–2012. PLoS One. (2016) 11(1):e0147923. 10.1371/journal.pone.0147923 26812646 PMC4728114

[130] Barua A Watson K Plesons M Chandra-Mouli V Sharma K. Adolescent health programming in India: a rapid review. Reprod Health. (2020) 17:87. 10.1186/s12978-020-00929-4 32493471 PMC7271491

[131] Taneja G Sridhar VSR Mohanty JS Joshi A Bhushan P Jain M India's RMNCH+A strategy: approach, learnings, and limitations. BMJ Glob Health. (2019) 4:e001162. 10.1136/bmjgh-2018-001162 31139464 PMC6509590

[132] Mai M Hassen E Ntabona AB Bapura J Sarathy M Yodi R Government ownership and adaptation in scale-up: experiences from community-based family planning programme in the democratic republic of the Congo. Afr J Reprod Health. (2019) 23(4):35–45. 10.29063/ajrh2019/v23i4.5 32227738

[133] Keyonzo N Nyachae P Kagwe P Kilonzo M Owino K Kichamu G From project to program: tupange's Experience with scaling up family planning interventions in urban Kenya. Reprod Health Matters. (2015) 23(45):103–13. 10.1016/j.rhm.2015.06.010 26278838

[134] Igras S Sinai I Mukabatsinda M Ngabo F Jennings V Lundgren R. Systems approach to monitoring and evaluation guides scale up of the standard days method of family planning in Rwanda. Glob Health Sci Pract. (2014) 2(2):234–244. 10.9745/GHSP-D-13-00165 25276581 PMC4168622

[135] Ntabona A Binanga A Bapitani MDJ Bobo B Mukengeshayi B Akilimali P The scale-up and integration of contraceptive service delivery into nursing school training in the democratic republic of the Congo. Health Policy Plan. (2021) 36(6):848–860. 10.1093/heapol/czab014 34009259 PMC8227455

[136] Chitama D Baltussen R Ketting E Kamazima S Nswilla A Mujinja PGM. From papers to practices: district level priority setting processes and criteria for family planning, maternal, newborn and child health interventions in Tanzania. BMC Women's Health. (2011) 11(1):46. 10.1186/1472-6874-11-46 22018017 PMC3217841

[137] Shilton S Chandra-Mouli V Paul S Denno DM. Facilitators and barriers in the utilization of World Health Organization's Preventing early pregnancy guidelines in formulating laws, policies and strategies: what do stakeholders in Ethiopia say? Int J Adolesc Med Health. (2021) 33(5):20190028. 10.1515/ijamh-2019-0028 31271553

[138] Lipsky AB Gribble JN Cahaelen L Sharma S. Partnerships for policy development : a case study from Uganda's Costed implementation plan for family planning. Glob Health Sci Pract. (2016) 4(2):284–99. 10.9745/GHSP-D-15-00300 27353621 PMC4982252

[139] Gichane MW Mutesa M Chowa G. Translating evidence into policy change: advocacy for community-based distribution of injectable contraceptives in Zambia. Glob Soc Welf. (2019) 6(1):41–7. 10.1007/s40609-018-0115-y

[140] Thomas JC Reynolds HW Alterescu X Bevc C Tsegaye A. Improving referrals and integrating family planning and HIV services through organizational network strengthening. Health Policy Plan. (2016) 31(3):302–8. 10.1093/heapol/czv058 26135363 PMC6296328

[141] Faye S Johns B Baruwa E Ambrose K. Evaluating the costs and efficiency of integrating family planning services into HIV and AIDS treatment services in Zambia. Health finance and governance project. Bethesda, MD: Abt Associates (2015).

[142] Krug C Cavallaro FL Wong KLM Gasparrini A Faye A Lynch CA. Evaluation of Senegal supply chain intervention on contraceptive stockouts using routine stock data. PLoS One. (2020) 15(8):e0236659. 10.1371/journal.pone.0236659 32745110 PMC7398546

[143] Cavallaro FL Duclos D Cresswell JA Faye S Macleod D Faye A Understanding “missed appointments” for pills and injectables: a mixed methods study in Senegal. BMJ Glob Health. (2018) 3(6):e000975. 10.1136/bmjgh-2018-000975 30687521 PMC6326323

[144] Tawfik Y Rahimazai M Ahmadzai M Clark PA Kamgang E. Integrating family planning into postpartum care through modern quality improvement : experience from Afghanistan. Glob Health Sci Pract. (2014) 2(2):226–33. 10.9745/GHSP-D-13-00166 25276580 PMC4168614

[145] Milford C Scorgie F Rambally Greener L Mabude Z Beksinska M Harrison A Developing a model for integrating sexual and reproductive health services with HIV prevention and care in KwaZulu-Natal, South Africa. Reprod Health. (2018) 15(1):189. 10.1186/s12978-018-0633-1 30442150 PMC6238282

[146] Binanga A Bertrand JT. Pilot research as advocacy: the case of sayana press in Kinshasa, democratic republic of the Congo. Glob Health Sci Pract. (2016) 4(4):542–51. 10.9745/GHSP-D-16-00236 27979874 PMC5199173

[147] Byrne A Morgan A Soto EJ Dettrick Z. Context-specific, evidence-based planning for scale-up of family planning services to increase progress to MDG 5: health systems research. Reprod Health. (2012) 9:27. 10.1186/1742-4755-9-27 23140196 PMC3563623

[148] Spring H Datta S Sapkota S. Using behavioral science to design a peer comparison intervention for postabortion family planning in Nepal. Front Public Health. (2016) 4:123. 10.3389/fpubh.2016.00123 27446891 PMC4914549

[149] Burke HM Ambasa-Shisanya C. Evaluation of a communication campaign to improve continuation among first-time injectable contraceptive users in Nyando district, Kenya. Int Perspect Sex Reprod Health. (2014) 40(2):56–67. 10.1363/4005614 25051577

[150] Okigbo CC Speizer IS Corroon M Gueye A. Exposure to family planning messages and modern contraceptive use among men in urban Kenya, Nigeria, and Senegal: a cross-sectional study. Reprod Health. (2015) 12:63. 10.1186/s12978-015-0056-1 26199068 PMC4508879

[151] Beaudoin CE Chen H Agha S. Estimating causal effects with propensity score models: an evaluation of the touch condom Media campaign in Pakistan. J Health Commun. (2016) 21(4):415–23. 10.1080/10810730.2015.1095818 26855176

[152] Atagame KL Benson A Calhoun L Corroon M Guilkey D Iyiwose P Evaluation of the Nigerian Urban Reproductive Health Initiative (NURHI) program. Stud Fam Plann. (2017) 48(3):253–68. 10.1111/sifp.12027 28620974 PMC5896011

[153] Babalola S. Changes in ideational profiles of women of reproductive age in Urban Nigeria: the role of health communication. Health Educ Behav. (2017) 44(6):907–17. 10.1177/1090198117699510 28387572

[154] Kraft JM Oduyebo T Jatlaoui TC Curtis KM Whiteman MK Zapata LB Dissemination and use of WHO family planning guidance and tools: a qualitative assessment. Health Res Policy Syst. (2018) 16(1):42. 10.1186/s12961-018-0321-1 29789001 PMC5964918

[155] LeMay NV Bocock PJW. Building a national model for knowledge exchange in Malawi: findings from a health information needs assessment. J Health Commun. (2012) 17(Suppl 2):64–78. 10.1080/10810730.2012.666623 22724672

[156] Hargreaves JR Goodman C Davey C Willey BA Avan BI Armstrong JRM. Measuring implementation strength : lessons from the evaluation of public health strategies in low- and middle-income settings. *Health Policy and Planning* (2016) 31(7):860–7. 10.1093/heapol/czw001 26965038 PMC4977426

[157] Schellenberg J. Measuring implementation strength: literature review draft report. London: London School of Hygiene and Tropical Medicine (2012). Available at: https://ideas.lshtm.ac.uk/wp-content/uploads/2017/08/IDEAS-Measuring-implementation-strength-report.pdf (Accessed on July 27, 2021).

[158] Rowbotham S Conte K Hawe P. Variation in the operationalisation of dose in implementation of health promotion interventions: insights and recommendations from a scoping review. Implement Sci. (2019) 14:56. 10.1186/s13012-019-0899-x 31171008 PMC6555031

[159] Ridde V Pérez D Robert E. Using implementation science theories and frameworks in global health. BMJ Glob Health. (2020) 5(4):e002269. 10.1136/bmjgh-2019-002269 32377405 PMC7199704

[160] Nilsen P. Making sense of implementation theories, models and frameworks. Implement Sci. (2015) 10(1):1–13. 10.1186/s13012-015-0242-0 25895742 PMC4406164

[161] Phillips JF Jackson EF Bawah AA Macleod B Adongo P Baynes C The long-term fertility impact of the navrongo project in northern Ghana. Stud Fam Plann. (2012) 43(3):175–190. 10.1111/j.1728-4465.2012.00316.x 23185861

[162] Carmichael SL Mehta K Raheel H Srikantiah S Chaudhuri I Trehan S Effects of team-based goals and non-monetary incentives on front-line health worker performance and maternal health behaviours: a cluster randomised controlled trial in bihar, India. BMJ Glob Health. (2019) 4(4):e001146. 10.1136/bmjgh-2018-001146 31543982 PMC6730593

[163] Mutemwa R Mayhew S Colombini M Busza J Kivunaga J Ndwiga C. Experiences of health care providers with integrated HIV and reproductive health services in Kenya: a qualitative study. BMC Health Serv Res. (2013) 13:18. 10.1186/1472-6963-13-18 23311431 PMC3599716

[164] Myers A Sami S Onyango MA Karki H Anggraini R Krause S. Facilitators and barriers in implementing the Minimum initial services package (MISP) for reproductive health in Nepal post-earthquake. Confl Health. (2018) 12:35. 10.1186/s13031-018-0170-0 30127844 PMC6092803

[165] Tessema GA Gomersall JS Laurence CO Mahmood MA. Healthcare providers’ perspectives on use of the national guideline for family planning services in amhara region, Ethiopia: a qualitative study. BMJ Open. (2019) 9:e023403. 10.1136/bmjopen-2018-023403 30787080 PMC6398659

[166] Zulu JM Kinsman J Hurtig AK Michelo C George A Schneider H. Integrating community health assistant-driven sexual and reproductive health services in the community health system in Nyimba district in Zambia: mapping key actors, points of integration, and conditions shaping the process. Reprod Health. (2019) 16(1):122. 10.1186/s12978-019-0788-4 31409362 PMC6693243

[167] Awoonor-Williams JK Sory EK Nyonator FK Phillips JF Wang C Schmitt ML. Lessons learned from scaling up a community-based health program in the upper east region of northern Ghana. Glob Health Sci Pract. (2013) 1(1):117–33. 10.9745/GHSP-D-12-00012 25276522 PMC4168550

[168] Gold J Burke E Cisse B Mackay A Eva G Hayes B. Increasing access to family planning choices through public-sector social franchising: the experience of marie stopes international in Mali. Glob Health Sci Pract. (2017) 5(2):286–98. 10.9745/GHSP-D-17-00011 28655803 PMC5487090

[169] Adedini SA Babalola S Ibeawuchi C Omotoso O Akiode A Odeku M. Role of religious leaders in promoting contraceptive use in Nigeria: evidence from the Nigerian urban reproductive health initiative. Glob Health Sci Pract. (2018) 6(3):500–14. 10.9745/GHSP-D-18-00135 30287529 PMC6172128

[170] Rawlins BJ Kim YM Rozario AM Bazant E Rashidi T Bandazi SN Reproductive health services in Malawi: an evaluation of a quality improvement intervention. Midwifery. (2013) 29(1):53–9. 10.1016/j.midw.2011.10.005 22079625

[171] Agha S. The impact of a quality-improvement package on reproductive health services delivered by private providers in Uganda. Stud Fam Plann. (2010) 41(3):205–215. 10.1111/j.1728-4465.2010.00244.x 21469273

[172] Tran NT Yameogo WME Langwana F Gaffield ME Seuc A Cuzin-Kihl A Participatory action research to identify a package of interventions to promote postpartum family planning in Burkina Faso and the democratic Republic of Congo. BMC Women's Health. (2018) 18(1):1–10. 10.1186/s12905-018-0573-5 29976182 PMC6034289

[173] Chhoun P Kaplan KC Wieten C Jelveh I Lienemann M Tuot S Using participatory methods to build an mHealth intervention for female entertainment workers in Cambodia: the development of the mobile link project. Mhealth. (2019) 5:24. 10.21037/mhealth.2019.07.02 31559269 PMC6737450

[174] Butler N Johnson G Chiweza A Aung KM Quinley J Rogers K A strategic approach to social accountability: bwalo forums within the reproductive maternal and child health accountability ecosystem in Malawi. BMC Health Serv Res. (2020) 20:568. 10.1186/s12913-020-05394-0 32571301 PMC7310083

[175] Boydell V Neema S Wright K Hardee K. Closing the gap between people and programs: lessons from implementation of social accountability for family planning and reproductive health in Uganda. Afr J Reprod Health. (2018) 22(1):73–84. 10.29063/ajrh2018/v22i1.7 29777644

[176] Abuya T Njuki R Warren CE Okal J Obare F Kanya L A policy analysis of the implementation of a reproductive health vouchers program in Kenya. BMC Public Health. (2012) 12(1):540. 10.1186/1471-2458-12-540 22823923 PMC3490771

[177] Barden-O’Fallon J Mason J Tluway E Kwesigabo G Kamanyi E. Counseling on injectable contraception and HIV risk: evaluation of a pilot intervention in Tanzania. PLoS ONE. (2020) 15(4):e0231070. 10.1371/journal.pone.0231070 32243478 PMC7122807

[178] Nelson AR Cooper CM Kamara S Taylor ND Zikeh T Kanneh-Kesselly C Operationalizing integrated immunization and family planning services in rural Liberia: lessons learned from evaluating service quality and utilization. Glob Health Sci Pract. (2019) 7(3):418–434. 10.9745/GHSP-D-19-00012 31558598 PMC6816810

[179] Kim C Kirunda R Mubiru F Rakhmanova N Wynne L. A process evaluation of the quality improvement collaborative for a community-based family planning learning site in Uganda. Gates Open Res. (2019) 3:1481. 10.12688/gatesopenres.12973.2 31392298 PMC6650767

[180] Mayhew SH Sweeney S Warren CE Collumbien M Ndwiga C Mutemwa R Numbers, systems, people: how interactions influence integration. Insights from case studies of HIV and reproductive health services delivery in Kenya. Health Policy Plan. (2017) 32(suppl_4):67–81. 10.1093/heapol/czx097 PMC5886080 29194544

[181] Hasselback L Dicko M Viadro C Ndour S Ndao O Wesson J. Understanding and addressing contraceptive stockouts to increase family planning access and uptake in Senegal. BMC Health Serv Res. (2017) 17:373. 10.1186/s12913-017-2316-y 28549472 PMC5446687

[182] McCarthy OL Wazwaz O Osorio Calderon V Jado I Saibov S Stavridis A Development of an intervention delivered by mobile phone aimed at decreasing unintended pregnancy among young people in three lower middle income countries. BMC Public Health. (2018) 18(1):576. 10.1186/s12889-018-5477-7 29716571 PMC5930955

[183] Benson J Healy J Dijkerman S Andersen K. Improving health worker performance of abortion services: an assessment of post-training support to providers in India, Nepal and Nigeria. Reprod Health. (2017) 14:154. 10.1186/s12978-017-0416-0 29162119 PMC5696763

[184] Ayon S Jeneby F Hamid F Badhrus A Abdulrahman T Mburu G. Developing integrated community-based HIV prevention, harm reduction, and sexual and reproductive health services for women who inject drugs. Reprod Health. (2019) 16(S1):59. 10.1186/s12978-019-0711-z 31138238 PMC6538559

[185] Sprockett A. Review of quality assessment tools for family planning programmes in low- and middle-income countries. Health Policy Plan. (2016) 32(2):292–302. 10.1093/heapol/czw123 28207050

[186] Adongo PB Phillips JF Aikins M Arhin DA Schmitt M Nwameme AU Does the design and implementation of proven innovations for delivering basic primary health care services in rural communities fit the urban setting: the case of Ghana's community-based health planning and services (CHPS). Health Res Policy Syst. (2014) 12(1):16. 10.1186/1478-4505-12-16 24690310 PMC3994228

[187] Webster J Krishnaratne S Hoyt J Demissie SD Spilotros N Landegger J Context-acceptability theories: example of family planning interventions in five african countries. Implement Sci. (2021) 16(1):1–14. 10.1186/s13012-020-01074-z 33435959 PMC7805098

[188] Marston C Arjyal A Maskey S Regmi S Baral S. Using qualitative evaluation components to help understand context: case study of a family planning intervention with female community health volunteers (FCHVs) in Nepal. BMC Health Serv Res. (2020) 20(1):685. 10.1186/s12913-020-05466-1 32703196 PMC7379347

[189] Doyle AM Mulhern E Rosen J Appleford G Atchison C Bottomley C Challenges and opportunities in evaluating programmes incorporating human-centred design: lessons learnt from the evaluation of adolescents 360. Gates Open Res. (2019) 25:1472. 10.12688/gatesopenres.12998.1 PMC6635668 31363715

[190] Atchison CJ Mulhern E Kapiga S Nsanya MK Crawford EE Mussa M Evaluating the impact of an intervention to increase uptake of modern contraceptives among adolescent girls (15-19 years) in Nigeria, Ethiopia and Tanzania: the adolescents 360 quasi-experimental study protocol. BMJ Open. (2018) 8(5):e021834. 10.1136/bmjopen-2018-021834 29858422 PMC5988138

[191] Spring H Datta S Sapkota S. Using behavioral science to design a peer comparison intervention for postabortion family planning in Nepal. Front Public Health. (2016) 4:123. 10.3389/fpubh.2016.00123 27446891 PMC4914549

[192] Lauria ME Fiori K Jones HE Gbeleou S Kenkou K Agoro S Assessing the integrated community-based health systems strengthening initiative in northern Togo: a pragmatic effectiveness-implementation study protocol. Implement Sci. (2019) 14(1):92. 10.1186/s13012-019-0921-3 31619250 PMC6796416

[193] Aichatou B Seck C Baal Anne TS Deguenovo GC Ntabona A Simmons R. Strengthening government leadership in family planning programming in Senegal: from proof of concept to proof of implementation in 2 districts. Glob Health Sci Pract. (2016) 4(4):568–581. 10.9745/GHSP-D-16-00250 28031298 PMC5199175

[194] Karim AM Guichon D Yihun BY Zemichael NF Lorenzana K Barofsky J Application of behavioral economics principles to reduce injectable contraceptive discontinuation in rural Ethiopia: a stratified-pair, cluster-randomized field trial. Gates Open Res. (2019) 3:1494. 10.12688/gatesopenres.12987.2 32803127 PMC7416084

[195] Phiri S Feldacker C Chaweza T Mlundira L Tweya H Speight C Integrating reproductive health services into HIV care: strategies for successful implementation in a low-resource HIV clinic in Lilongwe, Malawi. J Fam Plann Reprod Health Care. (2016) 42(1):17–23. 10.1136/jfprhc-2013-100816 25902815 PMC4717379

[196] Tweya H Feldacker C Gugsa S Phiri S. Contraceptive use and pregnancy rates among women receiving antiretroviral therapy in Malawi: a retrospective cohort study. Reprod Health. (2018) 15:25. 10.1186/s12978-017-0440-0 29426333 PMC5807743

[197] Mulubwa C Hurtig AK Zulu JM Michelo C Sandøy IF Goicolea I. Can sexual health interventions make community-based health systems more responsive to adolescents? A realist informed study in rural Zambia. Reprod Health. (2020) 17(1):1. 10.1186/s12978-019-0847-x 31915022 PMC6950932

[198] Speizer IS Calhoun LM McGuire C Lance PM Heller C Guilkey DK. Assessing the sustainability of the Nigerian urban reproductive health initiative facility-level programming: longitudinal analysis of service quality. BMC Health Serv Res. (2019) 19:559. 10.1186/s12913-019-4388-3 31399085 PMC6688378

[199] Speizer IS Guilkey DK Escamilla V Lance PM Calhoun LM Ojogun OT On the sustainability of a family planning program in Nigeria when funding ends. PLoS ONE. (2019) 14(9):e0222790. 10.1371/journal.pone.0222790 31557217 PMC6762171

[200] Zulu JM Goicolea I Kinsman J Fossgard Sandoy I Blystad A Mulubwa C Community based interventions for strengthening adolescent sexual reproductive health and rights: how can they be integrated and sustained? A realist evaluation protocol from Zambia. Reprod Health. (2018) 15:145. 10.1186/s12978-018-0590-8 30153839 PMC6114497

[201] Azmat SK Ali M Hameed W Mustafa G Abbas G Ishaque M A study protocol: using demand-side financing to meet the birth spacing needs of the underserved in punjab province in Pakistan. Reprod Health. (2014) 11(1):39. 10.1186/1742-4755-11-39 24885657 PMC4059733

[202] Nilsen P Bernhardsson S. Context matters in implementation science: a scoping review of determinant frameworks that describe contextual determinants for implementation outcomes. BMC Health Serv Res. (2019) 19(1):189. 10.1186/s12913-019-4015-3 30909897 PMC6432749

[203] McCarthy OL Wazwaz O Jado I Leurent B Edwards P Adada S An intervention delivered by text message to increase the acceptability of effective contraception among young women in palestine: study protocol for a randomised controlled trial. Trials. (2017) 18(1):454. 10.1186/s13063-017-2191-1 28974258 PMC5627444

[204] McCarthy O Leurent B Edwards P Tokhirov R Free C. A randomised controlled trial of an intervention delivered by app instant messaging to increase the acceptability of effective contraception among young people in Tajikistan: study protocol. BMJ Open. (2017) 7(9):e017606. 10.1136/bmjopen-2017-017606 28939582 PMC5623472

[205] McCarthy OL Osorio Calderon V Makleff S Huaynoca S Leurent B Edwards P An intervention delivered by app instant messaging to increase acceptability and use of effective contraception among young women in Bolivia: protocol of a randomized controlled trial. JMIR Res Protoc. (2017) 6(12):e252. 10.2196/resprot.8679 29254910 PMC5748473

[206] Dwyer SC Ishaku SM Okunade F Reichenbach L Jain A. Feasibility of patent and proprietary medicine vendor provision of injectable contraceptives: preliminary results from implementation science research in Oyo and Nasarawa, Nigeria. Contraception. (2018) 98(5):460–2. 10.1016/j.contraception.2018.08.015 30145127

[207] Kim CR Kidula N Rammipi MK Mokganya L Gaffield ML. The Botswana medical eligibility criteria wheel: adapting a tool to meet the needs of Botswana's Family planning program. Afr J Reprod Health. (2016) 20(2):9–12. 10.29063/ajrh2016/v20i2.1 29553159

[208] Chambers DA Glasgow RE Stange KC. The dynamic sustainability framework: addressing the paradox of sustainment amid ongoing change. Implement Sci. (2013) 8(1):117. 10.1186/1748-5908-8-117 24088228 PMC3852739

[209] Foy R Sales A Wensing M Aarons GA Flottorp S Kent B Implementation science: a reappraisal of our journal mission and scope. Implement Sci. (2015) 10(1):51. 10.1186/s13012-015-0240-2 25928695 PMC4409721

