# Supplementary material for: Use of implementation science to advance family planning programs in low- and middle-income countries: A systematic review
Source: Front Glob Womens Health. 2022 Dec 6;3:1038297. doi: 10.3389/fgwh.2022.1038297 (PMC9763469; doi:10.3389/fgwh.2022.1038297)
Supplement: Supplementary file 1 [file Datasheet1.pdf]

Supplementary Material

1 Supplemental Table 1

S1: Systematic Review: Participants, Intervention, Comparison, Context, Outcomes (PICCO) (includes PROSPERO criteria on contexts and additional outcomes).

Participants	The types of human participants included in the primary studies that this review will examine are those who introduce and incorporate FP related EBI into utilizing IS:<br><br>• Delivery system actors: individuals, teams, and organizations in LMIC health systems that adopt and integrate FP-related EBI into practice.<br>• Support system actors: individuals, teams, and organizations in LMIC that build the capacity of the delivery system actors to adopt and integrate FP-related EBI.<br>• Synthesis and translation system actors: individuals, teams and organizations that research, identify, translate, and disseminate knowledge on FP-related EBI to promote their uptake and spread.
Intervention	In this review, we are not studying the efficacy or effectiveness of specific EBI or exposures. Rather, we are studying how the above 'participants' have used IS to advance FP in LMIC. The studies we examine will emphasize the following two facets of using IS:<br><br>• Implementation strategies: the design, introduction, integration, and facilitation of activities aimed at enhancing the adoption, implementation, and sustainability of an FP-related EBI.<br>• Implementation research: inquiries that draw upon applicable theory, adapt, and customize methods, and is embedded in, or is overlaid upon, the conduct of implementation strategies to generate knowledge on their processes, effects and/or effectiveness.
Comparison	This systematic review does not require comparison or control groups.
Contexts	Studies may be excluded from the third stage of the systematic review (see below) if during the full-text review analysts determine that the application of IS described in it does not sufficiently reflect the following "defining characteristics of implementation science employed in global health"<br><br>1. Contextualization of implementation strategy and research design and methods according to levels of analysis, their environments.<br>2. Relevance of the implementation strategy and research to addressing health problems, decision-making needs, setting priorities and/or building commitment and levels where implementation strategy is conducted.

<table>
<tr><td></td><td>
3. Research questions are framed or based on needs identified by consumers of the evidence, and research design, methods and data sources are appropriate for the underlying implementation and decision contexts.
4. Multi-stakeholder and multidisciplinary co-creation of implementation strategies, co-production of research and collaborative use of results.
5. Embeddedness of the research in ‘real world’ conditions, i.e., the reality of the implementing organizations and communities and in the context of health systems.
6. Research is designed to provide evidence or solutions to implementers through short feedback loops that can be used for real-time improvements.
7. Research focuses on processes and outcomes and seeks to explain the generative process whereby results were produced under certain circumstances.
</td></tr>
<tr><td>Outcomes</td><td>
Mainly, this systematic review will:

• Synthesize trends, including strengths and gaps, with respect to the operationalization of IS to advance FP in LMIC.
o How have IS concepts and constructs been incorporated and applied toward the design and execution of implementation strategies to promote EBI uptake?
o How have IS theory and methods been employed in the application of research on processes, effects and effectiveness of strategies aimed at promoting EBI uptake?
o How have the principles of IS (see ‘contexts’ section below) been emphasized during the combined applications of implementation strategies and research?

Render insight and lessons learned, and direct recommendations for the future applications of IS to facilitate better adoption, implementation, and sustainment of FP-related EBI in LMIC to readers from policy, research, donor and technical assistance realms.
</td></tr>
<tr><td>Additional outcomes</td><td>
The studies, themselves, will have reported outcomes and examining these will add value and help reinforce the main outcomes of this review. Accordingly, reviewers will examine eligible studies for characteristics of the outcomes that each reports:

1. Improve populations’ health.
2. Enhance policy design and implementation.
3. Strengthen health systems and scale up,
4. Improve management and service delivery.
5. Support and empower communities to learn and act.

This will illustrate trends– in the value by each study, in terms of the individual knowledge contribution and impact on populations and health systems broadly.
</td></tr>
</table>

2 Supplemental Table 2

S2: Search Criteria

Concept 1: Subject area

1. (sexual and reproductive health OR sexual health OR reproductive health OR family planning OR contraception)

AND

Concept 2: Implementation strategy

2. (intervention OR interventions OR intervene OR intervenes OR intervened OR intervening OR implement OR implements OR implemented OR implementation OR project OR program OR disseminate OR disseminates OR dissemination OR integrate OR integration OR integrated OR integrates OR capacity building OR capacity build OR build capacity OR scale up OR scale-up)

AND

Concept 3: Implementation outcomes

3. (acceptability OR accept OR acceptance OR accepted OR adoption OR adopt OR adopted OR adopts OR appropriateness OR appropriate OR cost OR costs OR cost-effectiveness OR economic OR coverage OR reach OR feasibility OR feasible OR practical OR practicable OR fidelity OR adherence OR adherent OR penetration OR penetrated OR penetrate OR sustainable OR sustainability OR sustain OR sustains OR effective OR effectiveness OR implementation science OR implementation research OR implementation outcome OR implementation outcomes OR dissemination OR disseminate OR dissemination research)

AND

Concept 4: Geographic setting (LMICs)

4. (Afghanistan[tw] OR Albania[tw] OR Algeria[tw] OR Samoa[tw] OR Angola[tw] OR Antigua[tw] OR Barbuda[tw] OR Argentina[tw] OR Armenia[tw] OR Azerbaijan[tw] OR Bangladesh[tw] OR Belarus[tw] OR Belize[tw] OR Benin[tw] OR Bhutan[tw] OR Bolivia[tw] OR Bosnia[tw] OR Herzegovina[tw] OR Botswana[tw] OR Brazil[tw] OR Bulgaria[tw] OR Burkina Faso[tw] OR Burundi[tw] OR Cambodia[tw] OR Cameroon[tw] OR Cabo Verde[tw] OR Central African Republic[tw] OR Chad[tw] OR Chile[tw] OR China[tw] OR Colombia[tw] OR Comoros[tw] OR Congo[tw] OR Costa Rica[tw] OR Côte d'Ivoire[tw] OR Cote d'Ivoire[tw] OR Ivory[tw] OR Cuba[tw] OR Djibouti[tw] OR Dominica[tw] OR Dominican[tw] OR Ecuador[tw] OR Egypt[tw] OR Salvador[tw] OR Eritrea[tw] OR Ethiopia[tw] OR Fiji[tw] OR Gabon[tw] OR Gambia[tw] OR Georgia[tw] OR Ghana[tw] OR Grenada[tw] OR Guatemala[tw] OR Guinea[tw] OR Guinea-Bissau[tw] OR Guyana[tw] OR Haiti[tw] OR Honduras[tw] OR India[tw] OR Indonesia[tw] OR Iran[tw] OR Iraq[tw] OR Jamaica[tw] OR Jordan[tw] OR Kazakhstan[tw] OR Kenya[tw] OR Kiribati[tw] OR

Korea [tw] OR Kosovo[tw] OR Kyrgyz [tw] OR Lao[tw] OR Laos[tw] OR Latvia[tw] OR Lebanon[tw] OR Lesotho[tw] OR Liberia[tw] OR Libya[tw] OR Lithuania[tw] OR Macedonia[tw] OR Madagascar[tw] OR Malawi[tw] OR Malaysia[tw] OR Maldives[tw] OR Mali[tw] OR Marshall[tw] OR Mauritania[tw] OR Mauritius[tw] OR Mexico[tw] OR Micronesia[tw] OR Moldova[tw] OR Mongolia[tw] OR Montenegro[tw] OR Morocco[tw] OR Mozambique[tw] OR Myanmar[tw] OR Namibia[tw] OR Nepal[tw] OR Nicaragua[tw] OR Niger[tw] OR Nigeria[tw] OR Pakistan[tw] OR Palau[tw] OR Panama[tw] OR Papua New Guinea[tw] OR Paraguay[tw] OR Peru[tw] OR Philippines[tw] OR Romania[tw] OR Russia[tw] OR Russian[tw] OR Rwanda[tw] OR Samoa[tw] OR Sao Tome[tw] OR Senegal[tw] OR Serbia[tw] OR Seychelles[tw] OR Sierra Leone[tw] OR Solomon Islands[tw] OR Somalia[tw] OR South Africa[tw] OR Sri Lanka[tw] OR St. Lucia[tw] OR St. Vincent[tw] OR Grenadines[tw] OR Sudan[tw] OR Suriname[tw] OR Swaziland[tw] OR Syrian[tw] OR Syria[tw] OR Tajikistan[tw] OR Tanzania[tw] OR Thailand[tw] OR Timor-Leste[tw] OR Togo[tw] OR Tonga[tw] OR Tunisia[tw] OR Turkey[tw] OR Turkmenistan[tw] OR Tuvalu[tw] OR Uganda[tw] OR Ukraine[tw] OR Uruguay[tw] OR Uzbekistan[tw] OR Vanuatu[tw] OR Venezuela[tw] OR Vietnam[tw] OR "West Bank"[tw] OR Gaza[tw] OR Yemen[tw] OR Zambia[tw] OR Zimbabwe [tw] OR developing countries[mh] OR "developing countries"[tw] OR "resource-limited"[tw] OR "resource-constrained"[tw] OR "low- and middle-income"[tw] OR LMIC[tw] OR "third world"[tw] OR "low income countries"[tw])

AND
Articles are published during and after 2007
